# Cellular Protein Quality Control in Diabetic Cardiomyopathy: From Bench to Bedside

**DOI:** 10.3389/fcvm.2020.585309

**Published:** 2020-10-15

**Authors:** Namrita Kaur, Rida Raja, Andrea Ruiz-Velasco, Wei Liu

**Affiliations:** Division of Cardiovascular Sciences, School of Medical Sciences, Faculty of Biology, Medicine, and Health, The University of Manchester, Manchester, United Kingdom

**Keywords:** diabetic cardiomyopathy (DCM), cardiovascular disease, protein quality control (PQC), autophagy, proteostasis, unfolded protein response, proteasome

## Abstract

Heart failure is a serious comorbidity and the most common cause of mortality in diabetes patients. Diabetic cardiomyopathy (DCM) features impaired cellular structure and function, culminating in heart failure; however, there is a dearth of specific clinical therapy for treating DCM. Protein homeostasis is pivotal for the maintenance of cellular viability under physiological and pathological conditions, particularly in the irreplaceable cardiomyocytes; therefore, it is tightly regulated by a protein quality control (PQC) system. Three evolutionarily conserved molecular processes, the unfolded protein response (UPR), the ubiquitin-proteasome system (UPS), and autophagy, enhance protein turnover and preserve protein homeostasis by suppressing protein translation, degrading misfolded or unfolded proteins in cytosol or organelles, disposing of damaged and toxic proteins, recycling essential amino acids, and eliminating insoluble protein aggregates. In response to increased cellular protein demand under pathological insults, including the diabetic condition, a coordinated PQC system retains cardiac protein homeostasis and heart performance, on the contrary, inappropriate PQC function exaggerates cardiac proteotoxicity with subsequent heart dysfunction. Further investigation of the PQC mechanisms in diabetes propels a more comprehensive understanding of the molecular pathogenesis of DCM and opens new prospective treatment strategies for heart disease and heart failure in diabetes patients. In this review, the function and regulation of cardiac PQC machinery in diabetes mellitus, and the therapeutic potential for the diabetic heart are discussed.

## Introduction

Diabetes mellitus is one of the fastest-growing health issues worldwide, and it is a major threat to cardiovascular health. In 2019, it was estimated that 463 million people had diabetes, a number predicted to reach 700 million by 2045 (1), and diabetes patients have a 2–5-fold increased risk of developing heart failure (2, 3). Diabetic cardiomyopathy (DCM) refers to the cardiac dysfunction and structural abnormalities subsequent to diabetes, and independent of coronary artery disease, hypertension, and valve malfunctions (4, 5). The systemic metabolic alterations caused by reduced insulin secretion, in type 1 diabetes mellitus (T1DM), or progressive insulin resistance, in type 2 diabetes mellitus (T2DM), constitute continuous cardiac stress that leads to the activation of numerous cellular responses. DCM is characterized by impaired cellular homeostasis, the progressive accumulation of reactive oxygen species (ROS), reactive nitrogen species (RNS) and advanced-end glycation products, organelle dysfunction, and chronic inflammation. Eventually, DCM promotes pathological myocardial remodeling, resulting in cardiac dysfunction. Clinically, diastolic dysfunction is the first manifestation of DCM, followed by systolic dysfunction in later stages, and, ultimately, heart failure (6, 7). However, a single anti-diabetes agent (e.g., metformin or fibrate) is unable to ameliorate multiple comorbid conditions. The combination of individual therapies is indispensable for T2DM patients with other complications, including DCM.

**Figure 1:**
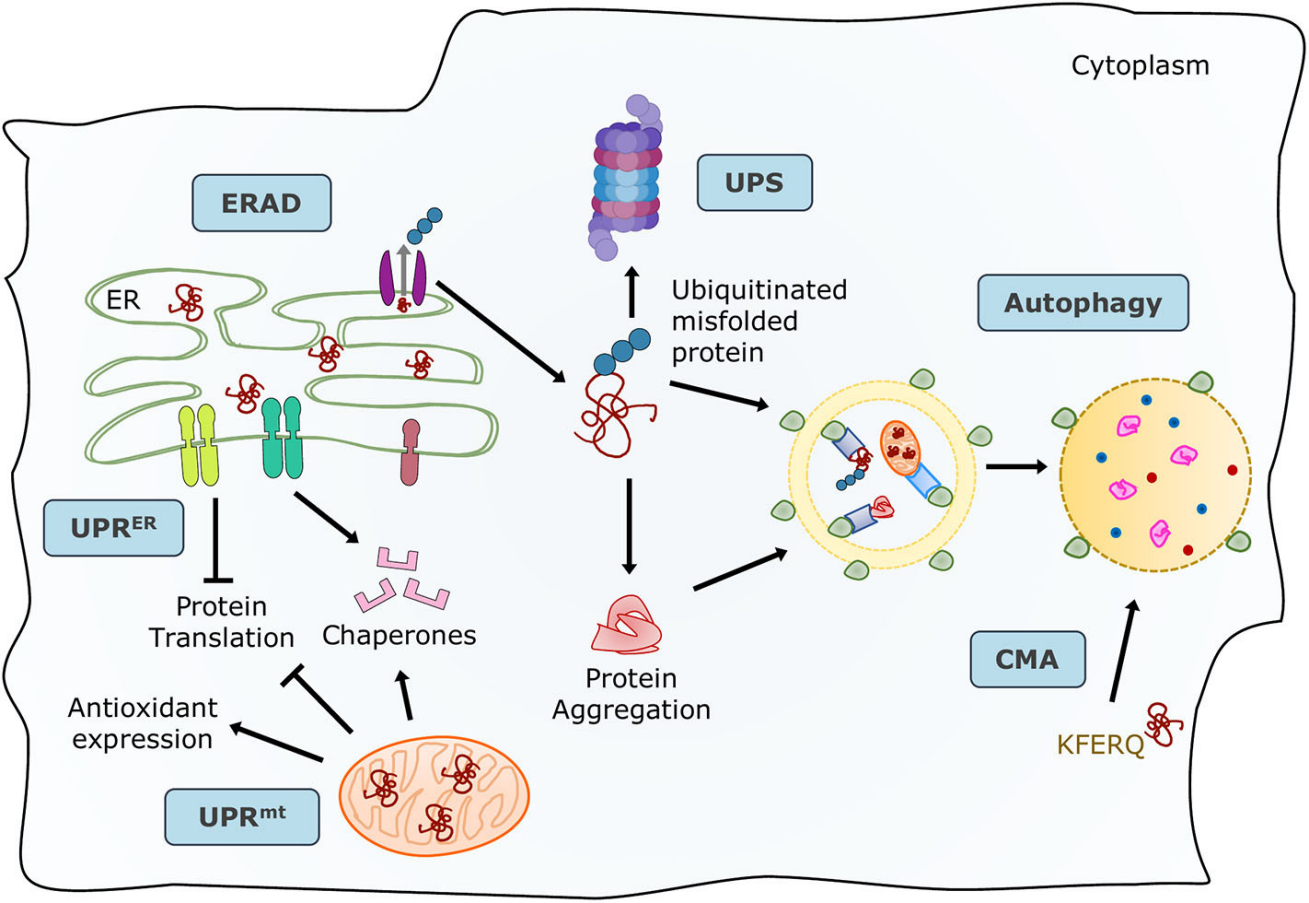
Maintenance of protein homeostasis by the principal PQC systems. Accummulation of misfolded proteins in the ER trigger the UPR to increase its folding capacity by upregulating chaperone expression, and to decrease protein load by inhibiting translation. Misfolded proteins are exported by ERAD complexes that label them and direct them to degradation through the UPS and autophagy. Misfolded or damaged proteins are also sequestered into protein aggregates to reduce their toxicity, these aggregates are processed by the autophagy-lysosome system. Proteins with the KFERQ motif are translocated into the lysosomes via CMA. Additionally, unfolded proteins in mitochondria induce the mtUPR to increase mitocondrial proteases and chaperones. Adapted from Ciechanover et al. (10) and used under CC BY 3.0.

Proteins are the primary managers of cellular homeostasis; therefore, regulation of their synthesis, maturation, and degradation in cardiomyocytes is essential for cardiac performance. To cope with the imbalance in the cardiac protein cycle in response to pathological stress, crucial protein quality control (PQC) systems participate in maintaining cellular protein homeostasis (8, 9) (Figure 1). Endoplasmic reticulum (ER) stress activates the unfolded protein response (UPR^ER^) to reduce protein synthesis, increase the expression of folding chaperones, and degrade non-functional proteins through the ER-associated protein degradation (ERAD) pathway. ERAD recognizes and translocates non-functional proteins into the cytosol for degradation. The ubiquitin-proteasome system (UPS) breaks down most proteins secreted by ERAD and those that have reached the end of their lifespan. Proteins that cannot be processed by the proteasome or protein aggregates are broken down via the autophagy-lysosome system. Similar to ER, mitochondria have a specific UPR (UPR^mt^) signaling to manage their unfolded protein load and can be selectively marked for autophagic degradation when the damage surpasses their coping capabilities (11). Coordination of PQC systems is adaptive and protective, while impaired PQC contributes to cardiac aging and diseases (12), including DCM (9). Therefore, it is crucial to comprehensively understand the function and regulation of PQC pathways to identify potential therapeutic targets and strategies for DCM.

## UPR

The ER is a central organelle for cellular PQC, operating as the keeper of the multistep maturation process of nascent polypeptides into functional proteins. The ER serves several cellular functions, comprising protein folding, posttranslational modifications, trafficking, calcium homeostasis, and lipid biosynthesis (13–16). Any intracellular and extracellular perturbations to its protein folding capacity result in ER stress and trigger the multi-faceted UPR^ER^ necessary for cellular PQC (17–20). Recently, mitochondria have been found to possess their own stress response to manage the unfolded proteins contained within them, also contributing to protein homeostasis (21).

### The UPR^ER^ Process

The primary intent of the UPR^ER^ is to adapt to any cellular changes by restoring protein homeostasis. The chaperones in the ER-lumen assist protein folding by binding to the hydrophobic regions of the nascent proteins (GRP78, GRP94), promoting glycoprotein interactions (Calreticulin, Calnexin), and facilitating the formation of disulfide bridges (ERP57, ERP78) (22). The master chaperone glucose regulatory protein 78 (GRP78) also binds to calcium, assists in ER permeability during protein translocation, and guides the misfolded proteins for degradation. The initial step of UPR^ER^ is the release of the transmembrane sensors, primarily bound to the master chaperone under non-stressed conditions (23). These UPR^ER^ sensors, including protein kinase RNA-like endoplasmic reticulum kinase (PERK), inositol-requiring enzyme 1 (IRE1), and activating transcription factor 6 (ATF6), are distinctively activated by stress stimuli and elicit varied adaptive downstream responses. Upon ER stress, PERK is majorly involved in attenuation of translation for lessening ER protein load via eukaryotic translation initiation factor 2α (eIF2α). PERK-eIF2α increases the expression of key genes facilitating UPR^ER^ via activating transcription factor (ATF4). On the other hand, the endoribonuclease activity of IRE1 causes splicing of the transcription factor, X-box binding protein 1 (XBP1). The spliced XBP1 (sXBP1) upregulates the expression of genes involved in UPR^ER^ signaling (9) and the ERAD pathway (24). IRE1-dependent decay (RIDD) is known to regulate essential ER-localized messenger RNAs (mRNAs) to reduce the inflow of newly synthesized proteins into the ER (25). IRE1 also enhances the degradation of terminally misfolded proteins via ERAD (UPS section) (18). Finally, upon activation, ATF6 translocates from the ER luminal domain to the Golgi apparatus, where site-1 and site-2 proteases cleave it to form an active segment, p50ATF6. The activated ATF6 transcriptionally regulates essential genes responsible for UPR^ER^ (26). All three UPR^ER^ branches are required to upregulate chaperones expression for assisting protein proper folding (27).

The ER stress response (ERSR) initially induces an adaptive UPR^ER^ to a certain threshold. In the face of chronic pathological stresses, oversaturated ER ensues apoptotic ERSR (28). Overexpression of ATF4 upregulates C/EBP homologous protein (CHOP), growth arrest, and DNA damage-inducible 34 (GADD34) and other pro-apoptotic genes (25). CHOP induces cell death by dysregulating the balance between pro- and anti-apoptotic genes from B-cell lymphoma 2 (BCL2) family. Also, oligomerization of the pro-apoptotic proteins BAX and BAK on the ER membrane causes calcium release into the cytosol, eventually promoting mitochondria-dependent apoptotic pathways (29). IRE1 induces ER-mediated apoptotic mechanisms via recruitment of TNF receptor-associated factor (TRAF) 2 and apoptosis signal-regulating kinase 1 (ASK1), leading to activation of c-Jun N-terminal kinase (JNK), and caspase-12 signaling pathways. In addition to IRE1-regulated caspase 12 cleavage, m-calpain, a cysteine protease, directly cleaves caspase-12 upon stimulation, resulting in its activation (30). The three UPR^ER^ branches exist to facilitate both cytoprotective and apoptotic responses depending on the nature of the stimulus (25, 27); therefore, it is not surprising that temporal dynamics of the UPR^ER^ has an important role in determining cellular fate.

### Physiopathological Role of UPR^ER^ in the Heart

ER-resident genes have been deemed essential in the heart. GRP78 or XBP1 deficiency is implicated in impaired cardiac development (31, 32) and cardiac dysfunction in response to pathological stresses (33, 34). The increase in protein disulfide isomerase (PDI) (35), an ER chaperone, and sXBP1 expression (36) in ischemic human hearts suggest UPR^ER^ is an adaptive component of the cardiac stress response. However, a maladaptive stress response is evident in dilated and failing human hearts marked by an increase in CHOP expression and cell death (37, 38). In light of the clinical evidence, it is apparent that the ERSR has both adaptive and maladaptive roles in cardiac pathology.

ER chaperones promote cell survival under pathological stress in the heart; nevertheless, overexpression is damaging. Cardiac GRP78 knockout in adult mice induced increased cell death, reduced cardiac performance, and caused early mortality (31). Moreover, the pre-induction of GRP78 and GRP94 had a cardioprotective role under oxidative damage in ischemia/reperfusion (39). On the contrary, increased protein synthesis under cardiac hypertrophy upregulated GRP78 expression, simultaneously, under pressure overload, GRP78 overexpression further potentiated hypertrophy by stimulating expression of hypertrophic factors resulting in cardiac dysfunction (40). Additionally, the overexpression of calreticulin, an ER chaperone, resulted in cardiac remodeling, dysfunction, and heart failure due to prolonged UPR^ER^ activation. This damaging effect of calreticulin overexpression *in vivo* was abated by inhibition of IRE1 (41, 42), overall suggesting the importance of balanced UPR^ER^ to tackle pathological stress in the heart.

Several animal studies targeting the individual UPR^ER^ branches emphasized the importance of UPR^ER^ in the pathological hearts of different etiologies. Cardiac PERK deficiency aggravated heart function in response to pressure overload in mice (43), indicating the cytoprotective role of the PERK branch. Moreover, transient IRE1-XBP1 response following pressure overload in mice (44) limited myocardial injury by reducing ER-associated cell death and inflammation (45) and promoting adaptive hypertrophy, in turn preserving contractility in hypertrophic failing hearts (25, 46). Similarly, cardiac XBP1 deficiency enhanced pathological remodeling and dysfunction (47). Lastly, ATF6 deletion in mouse hearts resulted in increased oxidative stress and decreased function after ischemia/reperfusion. The equivalent *in vitro* ATF6 knockdown model in cardiomyocytes showed similar results, which were obliterated by ATF6 overexpression (48). As noted, transient activation of all three UPR^ER^ branches has an adaptive function succeeding acute cardiac ER stress, while sustained activation of UPR^ER^ results in irreversible damage to the myocardium. This persistent stress signaling induces cardiomyocyte death via activation of ER-mediated apoptosis following myocardial infarction, ischemia/reperfusion, and pressure overload (23, 49). Also, Miyazaki et al. (50) demonstrated that cardiac CHOP deficiency inhibits ER-mediated myocardial apoptosis and inflammation following reperfusion injury, highlighting the role of maladaptive ERSR.

### The UPR^ER^ in DCM

#### Role of the UPR^ER^ and Apoptotic ERSR in DCM Development

The role of ER stress in the development of DCM was first observed in failing diabetic human hearts with swollen ERs (51), indicating protein imbalance. They also presented ER-mediated apoptosis, evidenced by increased CHOP and cell death (52). These clinical findings imply that the impaired UPR^ER^ predisposes the diabetic heart to failure; however, the precise nexus is elusive. The cardiac fate following ERSR has since been ascertained in several animal models of DCM. In the diabetic models, the elevation of cardiac ER stress-related markers (53) and UPR^ER^ genes (54–57) is associated with cardiac abnormalities (58) and apoptosis (59). Although the canonical UPR^ER^ signaling is an adaptive response, chronic ER stress is deleterious in the diabetic heart. In T1DM, prolonged ATF6 activation-induced cell death (60), extracellular matrix gene expression, cardiac fibrosis (61), and reduced cardiac compliance in rat models. Moreover, oxidative stress resulted in cardiac dysfunction in type 1 diabetic hearts via persistent PERK signaling (62). The role of over-activated PERK-CHOP and ATF6 ensuing apoptotic signaling via BCL2 associated agonist of cell death (BAD) and contributing to ER-mediated cardiac dysfunction was also recapitulated in T2DM rodent hearts (63). Apoptotic ERSR is associated with pathogenesis of DCM due to irreplaceable cardiomyocyte loss associated with the upregulation of cleaved-caspase 12, CHOP, and JNK in type 1 and type 2 diabetic hearts (51, 54). Altogether, the maladaptive ERSR in DCM prompts organelle dysfunction, cell death, and subsequent myocardial remodeling (64), suggesting that hyperactivated PERK and ATF6 are detrimental in DCM.

#### Metabolic Triggers of UPR^ER^ in DCM

**Figure 2:**
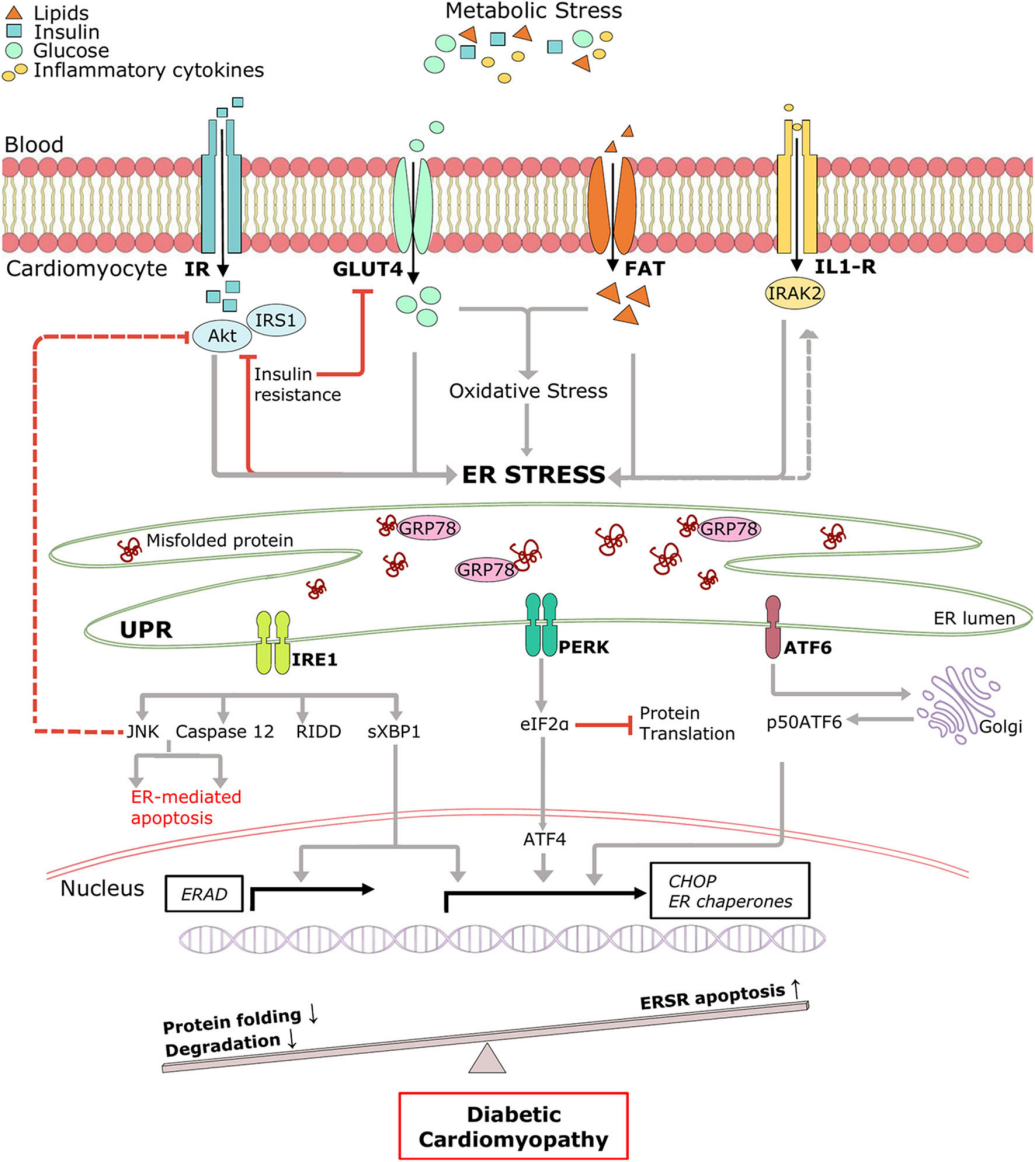
Pictorial representation of triggers of ER stress and role of ERSR activated UPR in the progression of DCM. Under systemic metabolic stress, lipid accumulation, and high glucose directly triggers ER stress and indirectly via oxidative stress. Hyperinsulinemia and inflammatory cytokines also induce ER stress, and in a feedback loop mechanism, ER stress triggers insulin resistance and inflammation (dotted lines). Upon accumulation of misfolded proteins, UPR signaling is activated following dissociation from GRP78. UPR sensors, IRE1, PERK, and ATF6 and its downstream network drives multiple signal outputs such as inhibition of protein translation and increased gene upregulation. Balanced UPR restores protein homeostasis by increasing ER chaperones and ERAD genes. Prolonged stress induces apoptotic ERSR via upregulation of CHOP, contributing to the pathogenesis of DCM. IR, Insulin receptor; IRS1, insulin receptor substrate 1; Akt, protein kinase B; GLUT4, glucose transporter type 4; FAT, fatty acid transporter; IL-R, Interleukin 1β receptor.

Pathological remodeling and cardiac dysfunction in DCM are accompanied by alterations in cellular protein synthesis, which can facilitate ER stress and UPR^ER^. ER stress is an early event in DCM, and the major triggers include hyperglycemia, hyperlipidemia, insulin deficiency/resistance, and inflammation (65, 66) (Figure 2). High glucose and lipid overload induce oxidative stress interceding dysregulated protein homeostasis, prolonged UPR^ER^, and cardiomyocyte death (67). Glucose and lipids upregulated adaptive IRE1-XBP1 signaling (51), and prolonged stress triggered apoptotic CHOP (63), IRE1-JNK (68), and caspase 12 activation in human cardiac cells (69), type 1 (55), and type 2 (30) diabetic rodent hearts. Inflammation and hyperinsulinemia are other factors that instigate ER stress and can be further potentiated by ER stress in a detrimental loop. As a coping mechanism, hyperinsulinemia-induced ER stress has emerged as a new player in the onset of insulin resistance (70, 71), possibly via IRE1/JNK signaling (58), contributing to reduced cardiac function in T2DM (72). On the other hand, pro-inflammatory cytokine interleukin-1β via interleukin 1 receptor-associated kinase 2 (IRAK2) promoted CHOP expression and cell death in T1DM, thereby impairing cardiac function (73). IRAK2 is known to be elevated in the condition of ER stress (74), suggesting a feedback loop mechanism accountable for unalleviated ER stress. However, the direct mechanism of ER-mediated inflammation and cardiac dysfunction in DCM is yet to be determined. Further mechanistic study of the intrinsic details of preferential UPR^ER^ under the numerous drivers of ER stress in DCM is essential.

#### Intrinsic Regulation of UPR^ER^ in DCM

**Figure 3:**
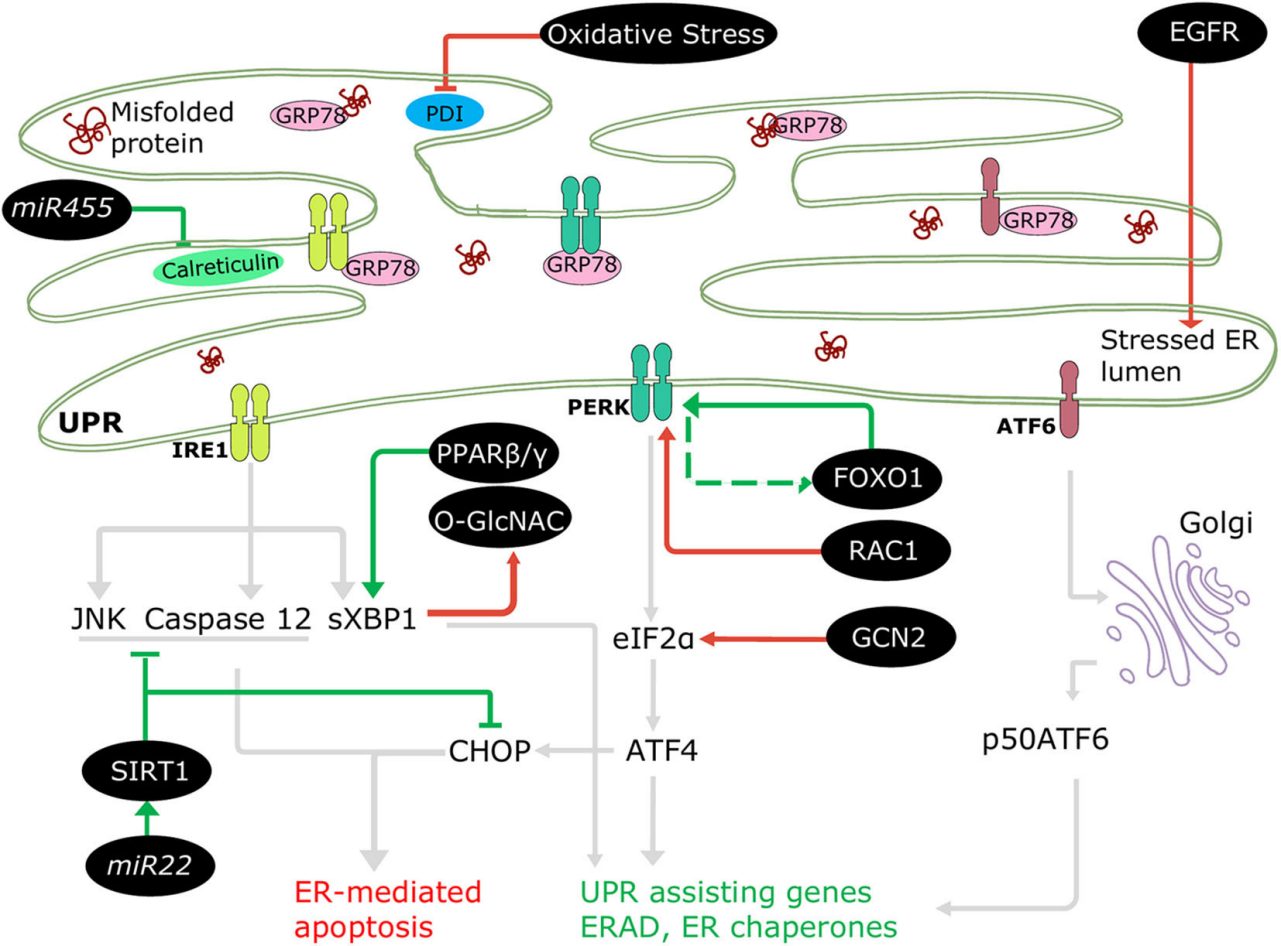
Regulation of ERSR in the diabetic heart. Some cellular molecules (FOXO1, PPARβ/γ, SIRT1, *mir22, mir455*) participate in regulation of adaptive ERSR by activating UPR and inhibiting ER-mediated apoptosis (green lines). On the other hand, some molecules and stresses (oxidative stress, O-GlcNAC, EGFR, RAC1, GCN2) stimulate maladaptive ERSR (red lines).

The involvement of ERSR in DCM progression is well-accepted; nonetheless, only a few regulatory mechanisms of UPR^ER^ in diabetes are documented, where the ER machinery coordinates with several cellular molecules and signaling pathways (Figure 3). For instance, downregulated NAD-dependent protein deacetylase sirtuin 1 (SIRT1) promoted stress signaling pathways such as IRE1-JNK in T1DM (75), and PERK-CHOP and IRE1-caspase 12 signaling in T2DM (76), resulting in ER-mediated apoptosis and cardiac dysfunction. A protein kinase, general control nonderepressible (GCN2), triggered cell death, and cardiac dysfunction directly via the eIF2α-ATF4-CHOP pathway in T1DM and T2DM (77). In addition, increased EGFR tyrosine kinase receptor activation instigated ER stress in T1DM (78) and in T2DM following myocardial infarction (79) by increasing CHOP associated cell death. The ERSR is also regulated via transcription factors. Forkhead box O1 (FOXO1) activation leads to direct and indirect induction of ER stress in DCM via PERK signaling (80, 81), and peroxisome-proliferator activator receptor (PPAR) β/γ activity promotes XBP1 splicing restoring ER balance and providing cryoprotection under diabetic stress in human cardiac cells (69). Additionally, microRNAs (miRNAs) have been observed to regulate UPR^ER^ in the diabetic heart. *mir455* and *mir22* are cardioprotective in T1DM (61) and T2DM (82), respectively. *Mir455* reduces cardiac fibrosis via calreticulin suppression, and *mir22* alleviates ER-mediated apoptosis via SIRT1 upregulation.

#### Role of Non-canonical UPR^ER^ in DCM

Apart from the regulated framework of UPR^ER^, diabetic condition also impairs UPR^ER^ capacity by directly regulating ER chaperones. Elevated PDI, despite the cardioprotective action under ischemic cardiomyopathy (35), was associated with increased cell death in hearts from diabetes patients (56). The lack of protective effect was attributable to the altered redox state of PDI under type 1 diabetes. Moreover, dual-color fluorescence imaging indicated an abnormal ER oxidative state and altered polarity in diabetic myocardial tissue (83), suggesting ER oxidative state may contribute to impaired UPR^ER^ under diabetes. Therefore, the maladaptive ERSR can be promoted by factors independent of canonical UPR^ER^ pathways in the diabetic heart. Also, post-translational modifications such as O-GlcNACylation are essential for protein stability and function. The protective effect of XBP1 on O-GlcNACylation (8, 46, 84) is absent in T2DM, leading to cardiac dysfunction (85), which is likely due to delayed UPR^ER^ action, as the timely UPR^ER^ lacks in the diabetic heart. Despite the growing knowledge about ERSR following various stresses faced by the diabetic heart, it is still unclear about how or when the switch between adaptive UPR^ER^ and apoptotic ERSR supervenes.

### Mitochondrial UPR (UPR^mt^) in the Heart

The mitochondrial proteome contains more than 1,300 proteins and the majority of the nuclear-encoded proteins are imported into the organelle in an unfolded state (86). Mitochondrial PQC entails protein import and folding via chaperones (HSP60, HSP70, and TRAP1) and degradation of misfolded proteins by proteases (ClpP, YME1L1, LonP1, HTRA2/Omi, and Oma1) (87). In response to stresses, the UPR^mt^ initiates a retrograde response to the nucleus to ensure proteome integrity via induction of the UPR^mt^-related chaperones (88). Akin to UPR^ER^, UPR^mt^ transiently inhibits protein translation and aims to mitigate proteotoxic stress inside the mitochondrion (89, 90). Under physiological conditions, activating transcription factor 5 (ATF5) is imported into the mitochondrion and degraded by LonP1; however, stress targets ATF5 to the nucleus as the transcription factor for the induction of UPR^mt^ (91). Owing to the cross-over among certain stress response proteins (PERK, ATF4, and CHOP) and the physical linkage between ER and mitochondria, both UPR^ER^ and UPR^mt^ participate in an integrated stress response to maintain cellular proteostasis (21). For instance, consequent to eIF2α activation, translation of ATF4, CHOP and ATF5 regulate the UPR^mt^. UPR^mt−^associated expression of CHOP is identified by binding of c-Jun to the AP-1 promoter region in the CHOP gene (92). CHOP binding, along with MURE1/2 elements in the promoter region, increases transcription of HSP60, ClpP, ATF5, and LonP1. Additionally, misfolded proteins are ubiquitinated in the inner mitochondrial space and degraded by the UPS in the cytosol in a process called mitochondrial associated degradation (93).

Mitochondrial PQC is essential for cardiac structure and function (94). In clinic, patients with ventricular pressure overload due to aortic stenosis had elevated ATF5 and reduced apoptosis (95), suggesting its protective role under cardiac stress. Analogous to clinical observation, silencing ATF5 in cardiomyocytes abated UPR^mt^ and its protection against pressure overload (96). Similarly, pharmacological stimulation of UPR^mt^ ameliorated cardiac dysfunction following ischemic injury via ATF5 induction (97). Moreover, HSP70 overexpression increased the import of antioxidant proteins, reduced cell death, and improved cardiac function against ischemic stress (98). As such, UPRmt is protective under cardiac stress; nonetheless, the role of mitochondrial proteases is still unclear. For instance, under hypoxia, mitochondrial protease LonP1 contributed to ROS accumulation and cell death in cultured cardiomyocytes (99). On the other hand, LonP1 overexpression was found to be protective following ischemic/reperfusion injury in mouse hearts (100) while reduced LonP1 activity in mitochondria contributed to contractile dysfunction after pressure overload (101). Interestingly, the same study demonstrated that LonP1 activation induces UPR^ER^; however, UPR^ER^ is activated before UPR^mt^, suggesting a fine-tuning role of LonP1 in the integrated stress response. More importantly, LonP1 deficiency was compensated via ATF4-dependent fibroblast growth factor (FGF21) activation (92, 101), a marker for mitochondrial stress signaling involved in mediating metabolic changes and ameliorating cardiac dysfunction under several cardiac etiologies, including diabetes (21, 102). Also, the mitochondrial protease, Oma1, is upregulated under cardiac ischemic stress; however, its ablation is protective against heart failure in mice (103).

UPR^mt^ chaperones are likely protective under diabetic stress in the heart. There is reduced expression of the mitochondrial chaperone, HSP70, in T2DM human hearts (104), indicating decreased protein import and UPR^mt^ induction. In the hearts of pre-diabetic rats with hyperinsulinemia, there was an increase in HSP60 expression; however, after prolonged diabetic stress HSP60-mediated myocardial protection decreased due to abated expression (105). Moreover, hyperglycemia reduced TRAP1 expression and activity, ultimately reducing cardiomyocyte viability (106). Furthermore, in T2DM rodent hearts, UPR^mt^ is responsible for the dysregulation of the mitochondrial permeability transition pore, associated with elevated cell death and ischemic injury (107). Overall, adaptive UPR^mt^ is critical for cardiac structure and function under diabetic stress; however, the detailed mechanistic role of the mitochondrial PQC and its therapeutic applications is yet to be cemented in DCM.

## UPS

The UPS is a major quality control pathway in eukaryotic cells, which plays a fundamental role in maintaining cellular proteostasis and, as such, ensures cell viability and function. The UPS is the primary proteolytic path for ~80% of cellular proteins, most of which are short-lived, misfolded, or damaged (108, 109). Mechanistically, the ubiquitin proteolytic pathway involves two distinct steps: ubiquitylation of protein substrates and degradation of the ubiquitylated proteins by the proteasome (110).

### UPS Process

#### Ubiquitylation

**Figure 4:**
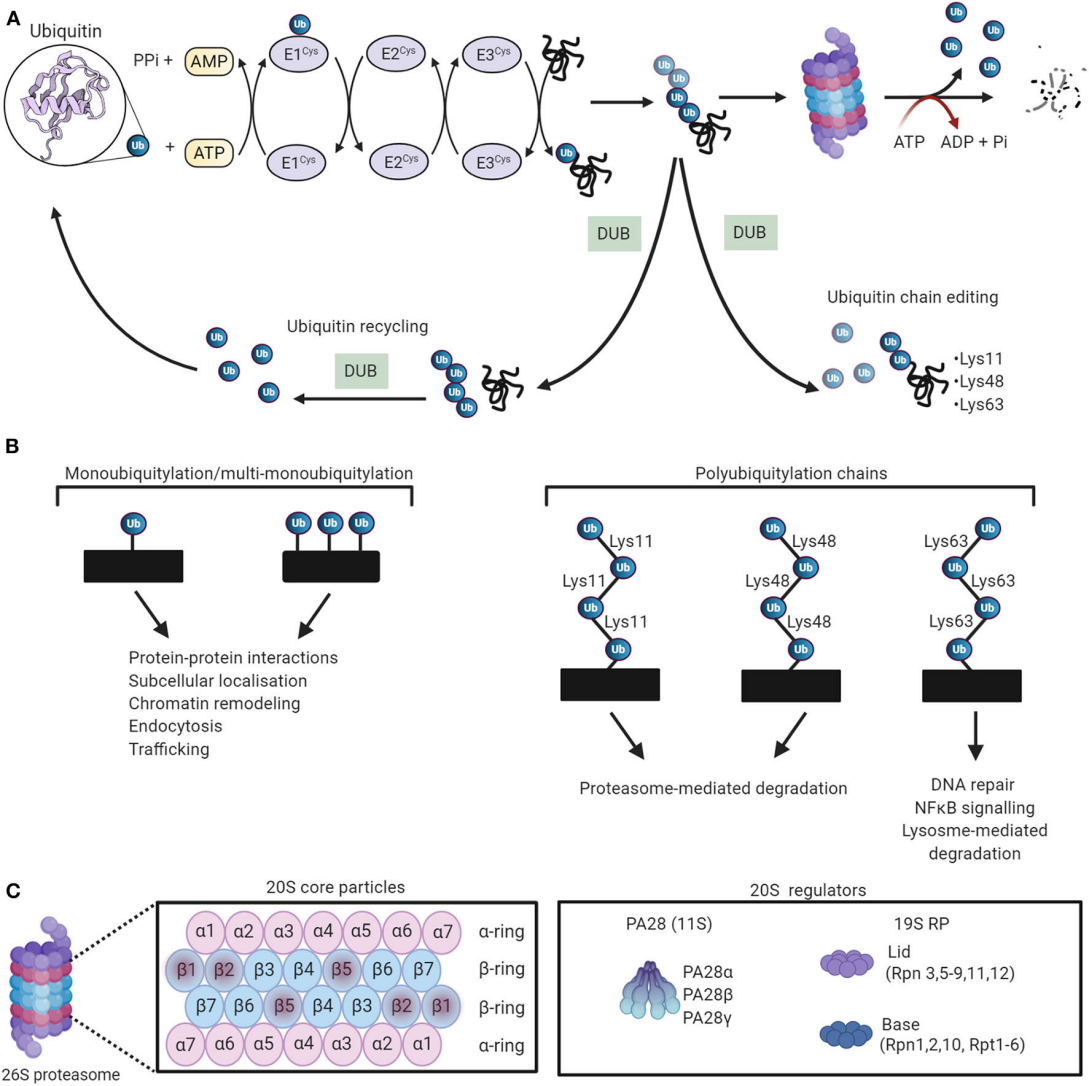
The ubiquitin-proteasome system (UPS) working theory. **(A)** The UPS marks substrate proteins for degradation via a ubiquitylation system where internal lysine residues of substrate proteins are tagged with ubiquitin (PDB ID: 1UBQ) (114). These ubiquitylated proteins are then degraded by the proteasome. Deubiquitylating enzymes (DUBs) edit ubiquitin chains and mediate ubiquitin recycling. Adapted from Zheng et al. (115) and used under CC BY. **(B)** The different ubiquitin linkages and their unique biological functions. **(C)** The proteosomal 20S core particle (CP) consists of four stacked rings, two outer rings composed of seven α subunits, and two inner rings composed of seven β subunits. The proteolytically active sites are localized in the β1, β2, and β5 subunits. The majority of 20S proteasomes are capped with 19S regulatory particles. The 20S can also be activated by PA28. Created with Biorender.com.

Ubiquitin is a 76-amino acid globular protein that is highly conserved in eukaryotes, and its transfer to target proteins is mediated by a carefully choreographed enzymatic cascade. Initially, ubiquitin is activated to a high-energy thiol ester state by the ubiquitin-activating enzyme E1 in an ATP-dependent manner. Following activation, the ubiquitin moiety is transferred to ubiquitin-conjugating proteins E2 by transesterification. Finally, an E3 ubiquitin ligase catalyzes ubiquitin transfer from the E2-ubiquitin thioester intermediate to a lysine residue on the substrate protein (111–113) (Figure 4A). The human genome encodes ~1,000 E3 ubiquitin ligases, which are subdivided into three major groups, depending on which of the following three domains they possess, namely, really interesting new gene (RING), RING-in-between-RING (RBR), and homologous to the E6-AP carboxyl terminus (HECT). It has been widely reported that the E3 ubiquitin ligases confer specificity to the ubiquitylation process (116). The proteins are targeted by either a single ubiquitin molecule (monoubiquitylation) or ubiquitin chains (polyubiquitylation). To date, eight structurally and functionally distinct ubiquitin linkages (Lys6, Lys11, Lys27, Lys29, Lys33, Lys48, Lys63, and Met1) have been identified, among of them, Lys48 and Lys63 are the most prominent linkage types (117–119). Lys48-ubiquitylated proteins are typically subjected to proteasomal degradation, while Lys63-linked ubiquitin chains mediate autophagic protein quality control (120, 121) (Figure 4B).

Akin to other posttranslational modifications, the ubiquitylation process is reversible; removal of ubiquitin molecules from substrate proteins is mediated by deubiquitylating enzymes (DUBs) (122). DUBs perform critical roles in the ubiquitylation pathway (123). First, *de novo* ubiquitin is translated as either linear polyubiquitylated chains, or ubiquitin fused to small ribosomal proteins, and DUBs are required to free mono ubiquitin from these precursors. Second, in consort with the E3 ligases, DUBs mediate ubiquitin chain editing, which can alter the ubiquitin signal or protein stability. Finally, DUBs maintain ubiquitin homeostasis by recycling ubiquitin molecules (122, 123).

#### Proteasomal Degradation

The degradation of polyubiquitylated proteins is catalyzed by the 26S proteasome, a large ATP-dependent multicatalytic complex composed of a barrel-shaped 20S core protease (CP) capped at one or both ends by the 19S regulatory particle (RP) (124) (Figure 4C). The 20S CP is composed of 28 subunits that are arranged as a cylindrical stack containing four hetroheptameric rings, two peripheral α-rings (α_1−7_), and two inner β-rings (β_1−7_). The two β-rings form the central proteolytic chamber, whereas the α subunits guard substrate entry into the chamber, impeding access when the proteasome is in an inactivated state (125, 126). The proteolytic activity of the 20S CP is activated by binding to the 19S RP to establish the proteasome holoenzyme (127). Protein components of the 19S RP recognize ubiquitylated substrates and transport them to the proteolytic core in an ATP-dependent manner (128–131). The peptidase activity of the 20 CP is also activated by other regulatory particles such as the 11S RP, which mediates protein degradation via a ubiquitin- and ATP-independent manner (132). The output of proteasomal degradation is small peptides, which, upon proteasomal exit, are further degraded by a plethora of cytosolic peptidases to generate amino acids to be recycled.

#### Chaperone-Assisted Proteasomal Degradation (CAP)

Molecular chaperones are essential for the folding fidelity and conformational integrity of proteins (133, 134), by participating in nascent polypeptide folding, protein transport, assembly of oligomeric complexes, and repair of misfolded proteins (133). In addition, chaperones can also facilitate the degradation of folding-incompetent proteins, thereby preventing their aggregation (133, 135). The C-terminus of Hsc70-interacting protein (CHIP) is a central player in chaperone-mediated degradation (136, 137). CHIP binds with the constitutively expressed HSP70/HSC70 chaperones and members of the ubiquitin conjugating enzyme, such as the Ubc4/5 family, to initiate chaperone substrate sorting to the proteasome or lysosome (135, 138). BAG family molecular chaperone regulator 1 (BAG1) is a co-chaperone which functions as a nucleotide exchange factor triggering ADP dissociation from HSP70/HSC70 proteins and thereafter promoting chaperone substrate release (139). Interestingly, BAG1 can also simultaneously bind to the proteasome via its Ub-like (UBL) domain thereby providing a functional link between chaperones and the proteolytic machinery (140). Conversely, the co-chaperone HSPBP1 attenuates CHIP ubiquitin ligase activity when it is complexed with HSP70/HSC70 and thus inhibits CHIP-mediated degradation (141). Notably, both CHIP and BAG1 exert cytoprotective effects in the heart following ischemia-reperfusion injury (142, 143).

In addition to CHIP and HSP70, chaperones such as HSP20, HSP90, and αB-crystallin (CryAB) are also induced in cardiomyocytes in an effort to buffer misfolded proteins during cardiac stress (137). Numerous studies have highlighted the protective role of these proteins in the heart (137). For instance, HSP90 appears to be cardioprotective in both doxorubicin-induced heart failure and high-glucose induced cell injury (144, 145). Moreover, cardiac specific over-expression of HSP20 attenuates apoptosis, reduces infarct size, and improves cardiac function in mice following ischemia-reperfusion injury (146). Mutations which impair the function of chaperones have been implicated in numerous diseases including cardiomyopathies (147). Pre-clinical studies have demonstrated that transgenic mice expressing an R120G-missense mutation in CRYAB develop restrictive cardiomyopathies and manifest pathological characteristics similar to those observed in clinical desmin-related myopathy (DRC); aberrant protein aggregation in cardiomyocytes and cardiac dysfunction (148, 149).

#### ERAD

ERAD is an integral facet of the UPS pathway (150). It is an evolutionarily conserved PQC mechanism in mammalian cells that orchestrates the function of numerous proteins to maintain ER homeostasis (151, 152). Through ERAD, aberrant ER luminal and transmembrane proteins are recognized and retrotranslocated to the cytosolic face where they are modified by the ubiquitylation machinery. The E3 ligases implicated in ERAD include soluble proteins, such as PRKN, ubiquitin conjugation factor E4 A (UBE4A), and CHIP, and ER transmembrane proteins, such as synoviolin (also known as HRD1), TEB4, GP78, and RMA1 (150). ERAD substrates are commonly conjugated to Lys48- and Lys11-linked polyubiquitin chains (153). Once ERAD substrates are adequately ubiquitylated, they are extracted from the ER membrane into the cytosol by the p97-UFD1-NPL4 complex to facilitate their proteasomal degradation (154). As such, if this adaptive ERAD function is defective or insufficient, the UPR^ER^ activates destructive cell pathways by transforming into an alternative signaling platform known as the terminal UPR^ER^ (155–158).

### The UPS in Cardiac Physiopathology

UPS activity is imperative in the heart as cardiomyocytes are highly susceptible to protein damage due to their constant exposure to metabolic and mechanical stress (159). Additionally, as terminally differentiated cells, cardiomyocytes possess minimal replicative potential; thus, failure to eliminate damaged proteins triggers excessive apoptosis, which is detrimental to the heart. Over the past decade, numerous clinical and experimental studies have documented impaired proteasome function, accumulation of ubiquitylated proteins, and alterations in the expression of UPS components in diseased hearts (159–164). Highlighting the importance of proteasomal integrity, cardiac proteasome inhibition induces heart dysfunction, and pathological hypertrophy in a preclinical mouse model (165). The pharmacological impediment of proteasome activity also leads to maladaptive structural and functional changes in pig hearts, which are consistent with a hypertrophic cardiomyopathy phenotype (166). Similarly, genetic inhibition of cardiac 20S proteasome promotes cardiac dysfunction in mice during systolic overload (138). Moreover, use of proteasome inhibitors (bortezomib, carfilzomib, and ixazomib), as targeted chemotherapeutics, is related to cardiovascular adverse events, including congestive heart failure (167). Of note, perturbations in UPS function have also been documented in doxorubicin-induced cardiotoxicity (168–170).

Inhibition of UBE2V1, a member of the E2 protein family, reduces protein aggregation in a *CryAB*^*R*120*G*^-desmin related myopathy mouse model, improves cardiac function, and enhances survival *in vivo* (171). Likewise, it has been firmly established that E3 ligases play a significant role in the pathogenesis of heart diseases (Table 1). In a preclinical model of pressure-overload, MURF1 knockout mice displayed exacerbated cardiac hypertrophy in response to mechanical stress (176). Similarly, transgenic mice expressing mutations in *Trim63*, the gene encoding MURF1, develop cardiac hypertrophy (204). Moreover, *Chip*–/– mice challenged with ischemia-reperfusion injury were more prone to arrhythmias and had decreased survival rates (143). However, research on the pathological implications of DUBs in the heart is limited (Table 1). A recent study revealed that the expression of ubiquitin carboxyl-terminal hydrolase isozyme L1 (UCHL1) was increased in the cardiomyocytes of hypertrophic and failing hearts (199). Overexpression of UCHL1 exacerbates pressure-overload induced cardiac hypertrophy and dysfunction, which can be reversed by systemic administration of the UCHL1 inhibitor LDN-57444 in mice (199). These studies demonstrate the detrimental effects of UPS malfunction in the myocardium.

**Table 1:** The role of the ubiquitylation pathway in cardiovascular disease.

		**Cardiac hypertrophy**	**Ischemia-reperfusion injury**	**Diabetic cardiomyopathy**	**Heart failure**
Cardiac-specific E3 ligases	**MURF1**	Potentially cardioprotective (172)	Cardioprotective (173, 174)		Deleterious (175)
	**MURF2**	Dispensable (176)		Cardioprotective (177)	
	**MURF3**		Potentially Cardioprotective (178)	Cardioprotective (179)	
	**Atrogin1/MAFbox**	Cardioprotective (180)	Deleterious (181)		Potentially deleterious (182)
Non-cardiac specific E3 ligases	**TTRIM72 (MG53)**	Cardioprotective (183)	Cardioprotective (184, 185)	Deleterious (186)	Cardioprotective (187)
	**TRIM21**	Deleterious (188)			
	**MDM2**	Cardioprotective (189)	Cardioprotective (189)	Potentially cardioprotective (80, 190)	
	**c-CB1**		Deleterious (191)		Potentially deleterious (191)
ERAD-associated E3 ligases	**CHIP**	Cardioprotective (192)	Cardioprotective (143)		
	**Parkin**		Cardioprotective (193, 194)	Potentially Cardioprotective (195, 196)	
	**HRD1**	Cardioprotective (197)			
	**GP78**			Potentially deleterious (198)	
Deubiquitylating enzymes	**UCHL1**	Deleterious (199)			
	**CYLD**	Deleterious (200)			
	**A20**	Cardioprotective (201)	Cardioprotective (202)		
	**USP4**	Cardioprotective (203)			

Furthermore, Doroudgar et al. demonstrated that HRD1 plays an essential role in the adaptive ERSR in cardiomyocytes and that cardiac-specific overexpression of HRD1 preserves cardiac structure and function in a mouse model of pathological cardiac hypertrophy (197). Moreover, overexpression of Derlin3, a component of the ERAD retrotranslocation channel, enhances ERAD-dependent disposal of misfolded proteins, attenuates exorbitant ERSR, and reduces caspase activity in response to ischemia/reperfusion injury (205). Conversely, knockdown of Derlin3 impairs the clearance of misfolded ER proteins and augments ischemia-mediated cell death in cardiomyocytes (205). Collectively, these findings suggest that ERAD-associated UPS plays a crucial role in myocardial viability and underscore the importance of PQC mechanisms in the setting of cardiac injury.

### The UPS in DCM

#### The E3 Ubiquitin Ligases in DCM

**Figure 5:**
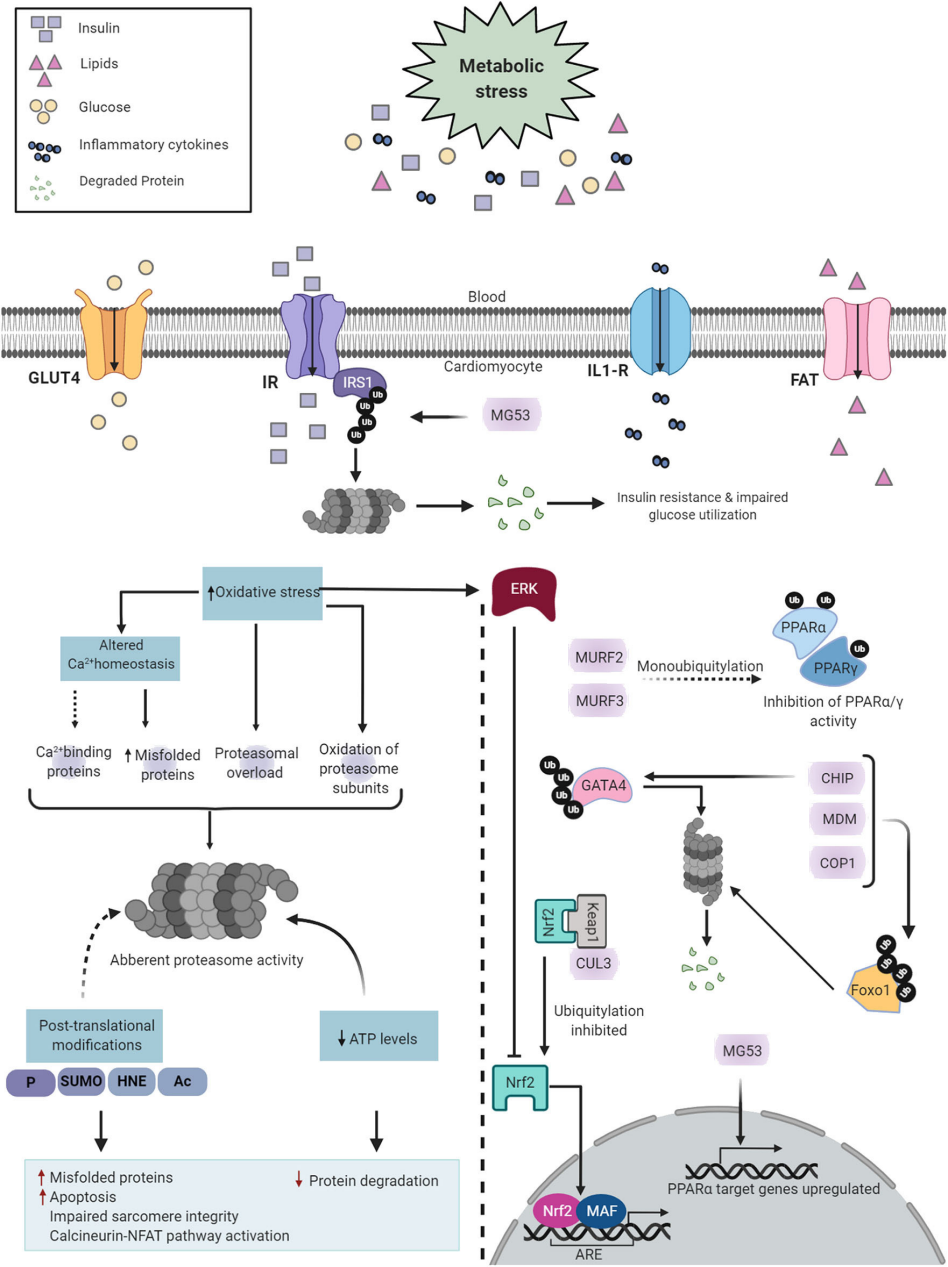
Diabetes-induced cardiac UPS dysfunction. Diabetes induces metabolic alterations in the heart that cause proteasome dysfunction in cardiomyocytes. Proteasome impairment may induce cardiomyopathy through multiple distinct mechanisms such as accumulation of misfolded proteins, enhanced apoptotic activity, contractile dysfunction and activation of calcineurin-NFAT pathway (Left). The E3 ligases regulate key transcription factors involved in DCM (Right). Created with Biorender.com.

The E3 ubiquitin ligases participate in cardiac metabolic regulation, by regulating numerous transcription factors involved in DCM (206) (Figure 5). FOXO1 has emerged as an influential player in the pathogenesis of DCM, which is overactivated in the hearts of murine models of T2DM. This aberrant activation is associated with the development of cardiomyopathy, evidenced by the cardiac-specific deletion of FOXO1 ameliorating high fat diet-induced cardiac dysfunction and preserved insulin responsiveness (80). At the molecular level, several E3 ubiquitin ligases, including CHIP, MDM, and COP1, regulate FOXO (190, 207, 208), as a consequence, the functions of FOXOs are subdued by virtue of their ubiquitin-mediated proteasomal degradation.

In addition, GATA4, a member of the GATA zinc-finger transcription factor family, is abundantly expressed in the heart. GATA4 regulates the transcription of numerous cardiac genes, including those involved in myocyte growth and survival (209). In both STZ-induced type 1 diabetic mice and *db/db* type 2 diabetic mice, GATA4 protein levels are significantly diminished in the heart (210), which is likely associated with the E3 ubiquitin ligase, CHIP (210).

Furthermore, PPARα is a member of the PPAR subfamily of nuclear receptors and acts as a prominent regulator of myocardial fatty acid utilization (211). Transgenic mice with cardiac-specific PPARα overexpression showed cardiac insulin resistance, reduced glucose utilization, lipid accumulation, and cardiomyopathy (212). MG53, an E3 ubiquitin ligase, and MURF1 are both regulators of PPARα (186, 213). Increased protein levels of MG53 results in a DCM-like phenotype (186). Mechanistically, not only does MG53 deteriorate insulin sensitivity, it also positively regulates PPARα, thereby inducing an energy source shift of glucose to fatty acid oxidation (186). Of interest, both MG53 and PPARα were also elevated in the hearts of *db/db* mice, HFD-induced obese mice, and rhesus monkeys with a spontaneous metabolic syndrome characterized by obesity, hyperlipidemia, and hyperglycemia (186). Moreover, MURF2 and MURF3 attenuate cardiac PPAR isoform activities and protect against DCM in HFD-challenged mice (177, 179).

Finally, the nuclear factor erythroid 2-related factor 2 (NRF2) is the master regulator of the cellular antioxidant response. NRF2 exerts transcriptional action on antioxidant genes through binding to the antioxidant response element (ARE), such as quinone oxidoreductase 1 (NQO1) (214), heme oxygenase-1 (HO1) (215), and superoxide dismutase 1 (SOD1). In addition to its antioxidant capabilities, NRF2 also enhances the clearance of toxic ubiquitylated proteins in the heart (216, 217). KEAP1, as an adaptor of the CUL3-RBX1 E3 ubiquitin ligase, binds NRF2, leading to its ubiquitylation and subsequent proteasomal degradation (218, 219). Human diabetic hearts show a significant reduction in NRF2 protein expression (220), associated with early-onset maladaptive cardiac remodeling and heart failure (220, 221). Both oxidative stress and misfolded proteins synergistically contribute to DCM; therefore, KEAP1 and the CUL3-RBX1 E3 ubiquitin ligase complex represent promising therapeutic targets for diabetic heart disease.

#### The Cardiac Proteasome in DCM

Diabetes induces both structural and functional alterations in the proteasome (Figure 5). In a recent study, Li et al. reported that STZ-induced diabetic mice exhibit a severe and progressive decline in cardiac proteasome activity, evidenced by a cumulative increase in GFPdgn (UPS function reporter) and Lys48-linked ubiquitylated protein levels in the heart (222). These alterations in proteasome activity precede the onset of cardiac dysfunction and thus could potentially play a pathogenic role in diabetic heart disease. In line with this, proteasome functional insufficiency was also reported in the myocardium of Sprague–Dawley rats subjected to T1DM, accompanied by higher levels of ubiquitylated and oxidized proteins (223). Taken together, these studies suggest that diabetes induces dissonance in proteasome activity and thereby distorts myocardial proteostasis.

Although the regulatory events underpinning these observations remain largely elusive, there are multiple mechanisms by which diabetes could lead to proteasome dysfunction, such as ATP depletion, oxidative stress, calcium imbalance, and diabetes-induced posttranslational modifications. ATP is required for both ubiquitin conjugation and the activation of the proteasome (224). Cardiomyocytes subjected to severe ATP depletion manifested profound proteotoxicity and stress-induced cell death (225). As such, reduced ATP levels, as observed in diabetic hearts (226–228), likely contribute to proteasome dysfunction.

Likewise, oxidative damage to proteasome subunits affects proteasome activity (229). Oxidation of the 19S regulatory particles Rpt3 and Rpt5 impairs the degradative capacity of the 26S proteasome (230). Bulteua et al. demonstrated that oxidation of the 20S proteasome also blocks proteasomal peptidase activity (231). Consistently, treatment with the NSAID meclofenamate sodium resulted in increased oxidative stress and concomitant oxidation of proteasome subunits, reducing proteasome activity (159, 232). Moreover, mitochondrial dysfunction-associated accumulation of 4-HNE, a secondary product of lipid peroxidation, directly inhibits the proteasome activity in failing rat heart (233). On the other hand, hyperglycemia-induced oxidative stress promotes aberrant cellular Ca^2+^ homeostasis (234, 235), subsequently leading to the accumulation of misfolded proteins and proteasomal overload. Altered cellular Ca^2+^ concentrations may influence the activity of the proteasome more directly by modulating the activity of Ca^2+^-binding proteins that interact with the proteasome. For instance, calmodulin binds to several non-ATPase subunits of the 26S proteasome and could alter proteasome activity (236).

Cardiac proteasome activity is influenced by posttranslational modifications, such as SUMOylation, glycosylation nitrosylation, and phosphorylation (237), which could be modified by the diabetic myocardial environment (222). Accordingly, protein kinase CβII (PKCβII), a classical PKC isoform, phosphorylates, and inhibits the proteasome activity in failing rat hearts (238). Treatment with a PKCβII inhibitor improves cardiac PQC, function, and survival (238). Abnormal proteasome activity compromises cardiac function through numerous mechanisms (239). Primarily, proteasomal derailment induces cardiac contractile inefficiency by impairing sarcomeres (239). Two parallel processes, assembly, and degradation, are necessary to maintain sarcomere integrity (175). The degradation of sarcomeric proteins is regulated almost exclusively by the UPS (240). Also, proteasome inhibition activates the calcineurin-NFAT pathway in the heart (241), which induces pathological hypertrophic growth (242). Finally, proteasome inhibition has been shown to induce apoptosis in cultured cardiac myocytes (243, 244).

## Autophagy

Autophagy is the homeostatic process through which cellular components are delivered to the lysosomes for degradation into their basic units. The cargo managed by the autophagic process includes insoluble and large misfolded proteins that cannot be degraded by the UPS (245), protein aggregates (246), and the proteasome itself (247). Autophagy can be triggered in the heart by various stress signals, such as nutrient deprivation, the absence of growth factors, and UPS malfunction (248).

### Autophagy Process

Autophagy comprises three types of processes (249). Macroautophagy (hereafter referred to as autophagy) requires the formation of double-membrane vesicles, named autophagosomes, to sequester cytoplasmic components and organelles. Fully developed autophagosomes are fused with the lysosomes, where lysosomal hydrolases break down all the elements contained, including the inner membrane. The second type, microautophagy, is when cytoplasmic components are engulfed and degraded through the invagination of the lysosomal membrane. The third type, chaperone-mediated autophagy (CMA), is the process through which proteins exposing a KFERQ motif are translocated into the lysosomes. Approximately 75% of the human proteome has potential KFERQ motifs (250). Even though autophagy and microautophagy are bulk processes engulfing everything in a section of the cytoplasm, they also function selectively. Organelle (251) and protein aggregate (252) labeling consists of ubiquitination, a process shared with the UPS (253). This label is recognized by autophagy receptors such as p62/sequestosome 1 (SQSTM1), BCL2/adenovirus E1B 19 kDa protein-interacting protein 3 (BNIP3), and BNIP3-like (NIX) (254). Selective autophagy is vital to prevent proteotoxicity and promote cellular survival.

**Figure 6:**
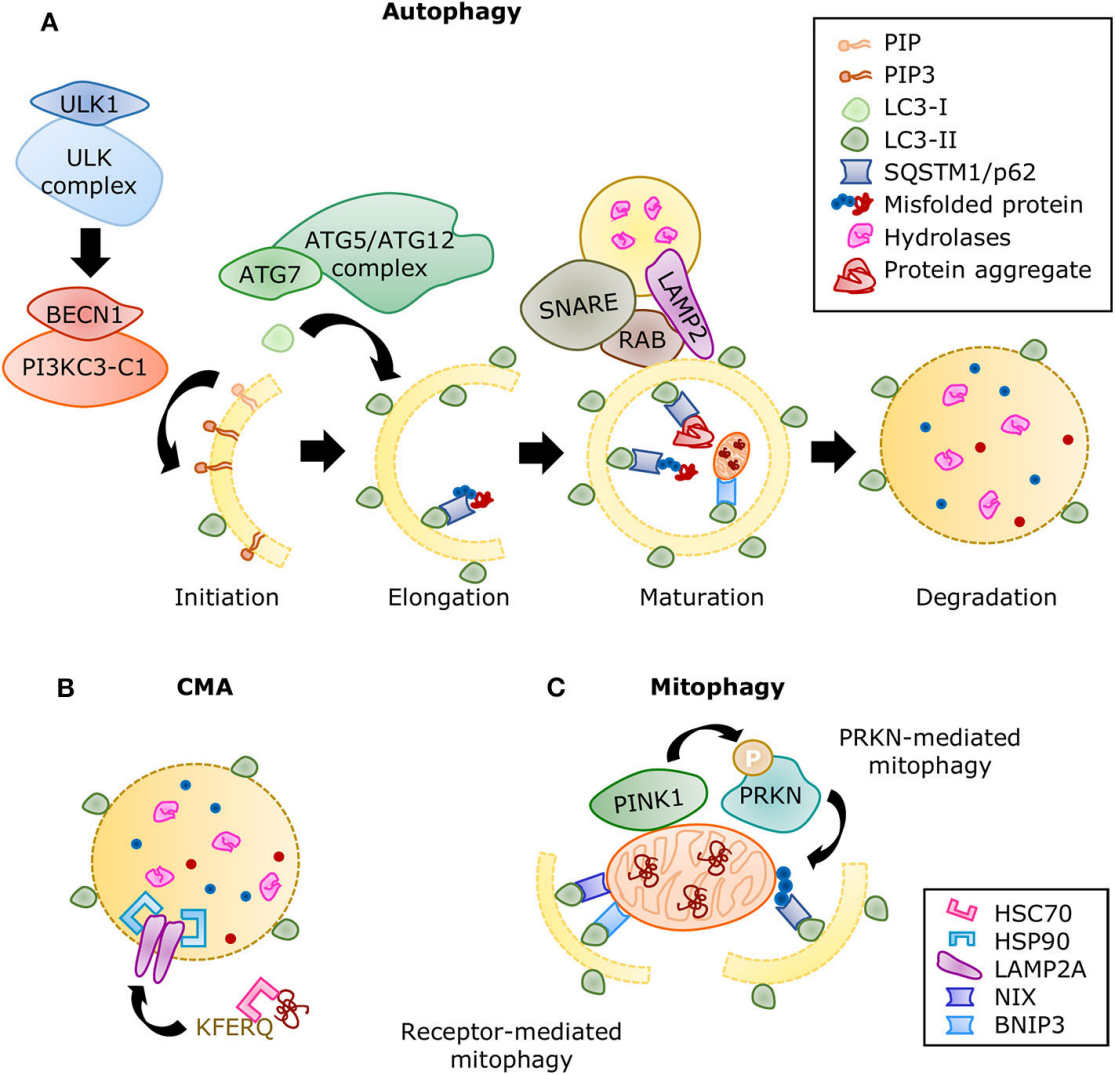
Summary of the autophagic processes. **(A)** A series of protein complexes drive autophagosome formation and fusion with the lysosome. The autolysosome breaks down misfolded proteins, protein aggregates, and organelles using a variety of hydrolases. Misfolded proteins are directed to selective autophagy through ubiquitination and detection by SQSTM1/p62. Adapted from Ciechanover et al. (10) and used under CC BY 3.0. **(B)** During CMA, proteins containing the KFERQ motif are recognized by the chaperone HSC70 and translocated directly into the lysosome by a complex of LAMP2A proteins stabilized with HSP90 chaperones. **(C)** Mitophagy is the selective degradation of nonfunctional mitochondria that have been labeled by the PINK1/PRKN system. Adaptor proteins such as SQSTM1 recognize the ubiquitinated proteins and linking it to the growing autophagosome. The receptor-mediated mitophagy is independent of PINK1/PRKN labeling, but directed by receptors, such as BNIP3 and NIX, which also contain LC3-binding domains.

The autophagic molecular machinery consists of numerous autophagy-related proteins (ATG), directing all the stages of autophagy: initiation, elongation, maturation, and degradation (Figure 6A). Initiation begins with ATG1, also known as ULK1, forming the serine/threonine-kinase ULK complex. This complex phosphorylates the class III phosphatidylinositol 3-kinase complex I (PI3KC3-C1) containing beclin 1 (BECN1). The latter complex produces phosphatidylinositol 3,4,5-trisphosphate (PIP3), initiating the formation of the phagophore. Elongation is driven by the lipidation of microtubule-associated proteins 1A/1B light chain 3 (LC3) by ATG7 and the ATG5/ATG12 complex, during which LC3-I is conjugated with phosphatidylethanolamine to form LC3-II. This process allows the incorporation of LC3-II into the membrane and stabilization of the phagophore. At this stage, the autophagy receptors recognize labeled components and bind to LC3-II. When the autophagosome is closed, small GTPases of the Ras-related protein in brain (RAB) family recruit tethering proteins to anchor the autophagosome and the lysosome together, while snap receptor (SNARE) proteins and lysosome-associated membrane glycoprotein 2 (LAMP2) regulate their fusion (255), this is the maturation to autolysosome. A variety of enzymes in the autolysosome carry out the degradation process, after which the macromolecules are released into the cytosol for anabolic reactions.

Molecular chaperones also have a significant role in all three types of autophagy mediating selectivity and stability of the processes. If protein refolding fails, they can also direct cargo for degradation. The function of chaperone heat shock cognate 71kDa protein (HSC70) was first described in CMA. HSC70 recognizes the KFERQ motif in proteins and facilitates their translocation into the lysosome through LAMP2A (249). A second chaperone, heat shock protein (HSP90) enhances binding of the substrates and LAMP2A stability (256) (Figure 6B). Furthermore, HSC70 was later associated with the targeting of cytosolic proteins toward endosomal microautophagy (257) and chaperone-assisted selective autophagy (CASA), both of which can manage the degradation of protein aggregates (258). The substrate and process specificity of chaperones participating in different types of degradation is believed to come from the formation of complexes with co-chaperones, whose availability depends on cell type and stress or stimulus conditions. For example, BAG3 is a co-chaperone that interacts with HSP70 and HSPB8 to trigger selective autophagy of aggregated proteins (259). Its counterpart, BAG1, guides proteins toward proteasomal degradation. Several other co-chaperones have been described (260); however, their specific part in cardiac function is still being explored.

### Physiopathological Role of Autophagy in the Heart

Basal autophagic activity in the myocardium is required to prevent the accumulation of misfolded proteins and the recycling of essential components from defective organelles to sustain cardiac function. Mutations in autophagy-related genes are the cause of dilated cardiomyopathies. The most common is Danon disease (261), a mutation of the *LAMP2* gene, characterized by the weakening of the heart, protein aggregation, accumulation of autophagic vesicles in the muscle, and cardiac hypertrophy. The multisystemic disorders rising from loss-of-function mutations in the *EPG5*(262), *PLEKHM2* (263), and *BAG3* (264–266) genes have significant cardiovascular manifestations and are the result of defective autophagy. On the contrary, inducing autophagy ameliorates desmin-related cardiomyopathy by clearing the protein aggregates that originate from the mutation of the *CRYAB* gene (267). Additionally, cardiomyocyte-specific ATG5 deletion in mice, since birth (268) and in the adult stage (269), caused systolic dysfunction and sarcomeric structure disarray without any further stress. Conversely, augmenting basal autophagy by ATG5 overexpression (270) or BECN1 mutation to prevent its association with BCL2 (271) increased longevity in mice. A certain capacity for autophagic processing is elementary for normal cardiac function, and its sustenance counteracts proteotoxicity.

Cardiac autophagic flux is strongly induced by fasting (272), caloric restriction (273), exercise (274), and in neonatal mouse hearts after the placental supply is interrupted at birth (275), which is pivotal for cardiac contractility and survival (276, 277). Fasting and caloric restriction stimulate autophagy in the heart through 5′-AMP-activated protein kinase (AMPK) phosphorylation (272, 273) and increased SIRT1-mediated FOXO1 activity (278). Upon nutrient restoration after birth, insulin and amino acids moderate autophagy. Insulin signaling inhibits autophagy through AKT serine/threonine kinase 1 (AKT)-mediated activation of the mechanistic target of rapamycin kinase complex 1 (MTORC1), an autophagy inhibitor. When postnatal autophagy inhibition was disrupted by genetic deletion of the insulin receptors, cardiomyocyte death, and heart failure occurred due to excessive autophagy (279). Unrestrained autophagy was diminished by the supplementation of amino acids, which suppressed autophagy (279) through RAG protein family regulation of MTORC1 (280). On the other hand, in exercised mouse hearts, AMPK activation promoted the expression of autophagic genes (274) and dissociated the BCL2/BECN1 complex to increase autophagy level (281). However, in the long-term, exercise increased the autophagic capacity by augmenting LC3 expression in cardiac muscle without boosting autophagic flux (282). Nevertheless, this increased capacity limited cardiac injury and improved function after myocardial infarction (283, 284). These observations suggest that the regulation of autophagy by exercise is multifaceted and adaptive.

The role of autophagy in cardiac disease has been more challenging to determine, since it can be adaptive or maladaptive depending on the specific pathology and pathogenesis stage. The presence of abnormal protein aggregation in the myocardium of dilated cardiomyopathy patients was associated with impaired cardiac autophagy (12), while the detection of autophagic vacuoles was associated with improved heart failure prognosis (285). In preclinical studies, myocardial ischemia-induced autophagy in mouse (286) and swine models (287), moderating apoptosis, and autophagy induction, in turn, limited myocardial injury (272, 288, 289). Autophagy was reduced after prolonged pressure overload in mouse hearts, and ATG5 deletion aggravated cardiac remodeling and performance (269). These results indicate that autophagy is required to preserve cardiac function in response to pathological stresses. However, excessive BECN1 expression and autophagosome formation were found to be detrimental during reperfusion (290) and pressure overload (291), also in mouse studies. The seemingly confusing outcomes could be explained by the discovery that BECN1 association to the Rubicon protein was able to inhibit autophagic flux by interfering with autophagosome maturation (292), which has been recently termed autosis (293), a form of cell death. In addition, substantial evidence indicate that maladaptive autophagy was observed in atrial fibrillation by the degradation of cardiac troponin I/T (294) and calcium channel CAV1.2 (295), resulting in contractility and electrical alterations. Collectively, adaptive autophagy is essential to cardiac health, whereas either insufficient or excessive autophagy is detrimental.

### Autophagy in DCM

The elucidation of the role of autophagy in DCM has been intricate due to the complexities of the disease and the duality of the nature of autophagy. In T2DM, autophagic flux is increased in the early stages of disease (296) and later reduced, with cardiac function improvement mostly being associated with therapeutic restoration of the autophagic flux (297–305). Increased expression of autophagy proteins was observed in human atrial samples and obese mouse myocardium, while fractional shortening was maintained (306). However, impaired autophagy by long-term chloroquine administration (300) or cardiac ULK1 deficiency was detrimental for cardiac function in obesity, resulting from fibrosis and apoptosis (297). On the contrary, boosting autophagic flux in a later stage by rapamycin administration improved systolic performance in high-fat diet (HFD)-induced diabetes (302). It has been suggested that autophagy contributes to high fructose-induced cardiomyopathy (307); nonetheless, these samples also displayed signs indicating that autophagic flux might have been blunted. In myocardial samples of obese and T2DM patients, amylin aggregates were detected and found to induce cardiac dysfunction (308). These aggregates, also known as islet amyloid polypeptide (IAPP) oligomers, disrupted autophagy-associated disposal, increasing their toxicity (309, 310). All together, autophagy is stimulated in T2DM stages with preserved cardiac function, while its abnormalities likely cause the onset and development of DCM and heart failure.

In T1DM models, the regulation and function of autophagy in the heart are elusive. Cardiac autophagy could be enhanced at an early time point (311); nonetheless, most evidence indicates it is suppressed. More importantly, preclinical experiments suggest that autophagy inhibition could be therapeutic in this case. Cardiac BECN1 overexpression in streptozotocin (STZ)-induced diabetes aggravated cardiac function (312). Conversely, autophagy reduction by BECN1 insufficiency and hypomorphic ATG16 improved echocardiographic measurements and hemodynamic analysis in the same model and in OVE26 mice, which develop severe early-onset T1DM due to deficient insulin production. Of note, even though autophagic flux was diminished, the functional improvements were accompanied by increased expression of RAB9 (312), which directed a non-canonical form of autophagy (313); therefore, it is speculated that non-canonical autophagy fulfills beneficial effects in T1DM. Akin to observations in T2DM patients (308), STZ-injected mice displayed toxic cardiac protein aggregation that can be improved by boosting autophagy (314). Interestingly, in T1DM and T2DM, CMA was found to be promoted even after autophagy was suppressed; however, evidence showed it could be contributing to metabolic inflexibility (7). As such, the molecular mechanisms underlying cardiac autophagy in DCM require further investigation.

**Figure 7:**
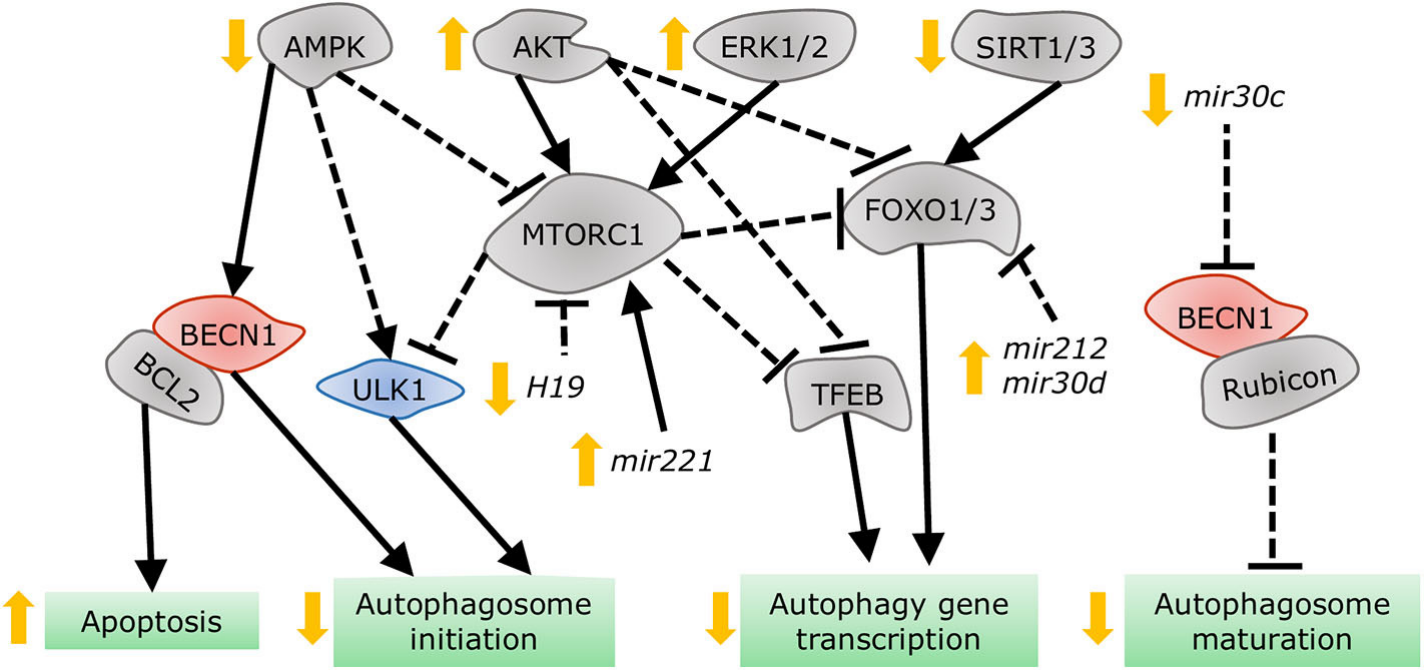
Dysregulation of autophagy in the diabetic heart. AMPK and SIRT1/3 inhibition, in addition to AKT and ERK1/2 stimulation, impair autophagy gene transcription, and autophagosome initiation via MTORC1, TFEB, and FOXO1/3 regulation. Decreased dissociation of the BECN1/BCL2 complex augments apoptosis, while increased association of BECN1 with Rubicon blunts autophagosome maturation. MiRNAs altered in diabetes interfere with autophagy by acting on players such as MTORC1, FOXO1/3, and BECN1.

Despite the distinct etiologies and biochemical stresses present in T1DM and T2DM, a few regulatory pathways have been demonstrated to be involved in autophagy suppression and the development of cardiac dysfunction (Figure 7). AMPK phosphorylation is reduced in the hearts of a number of DCM mouse models, including OVE26 (315), STZ-induced (316), diet-induced (302), and genetically obese mice. In OVE26 mice, the stimulation of AMPK phosphorylation by metformin restored autophagy and cardiac dysfunction (315). AMPK-mediated autophagy regulation is attained through numerous molecular mechanisms (316, 317), but mainly through repression of MTORC1 activity (302, 304, 318). MTORC1 suppresses autophagy by phosphorylating ULK1 (319, 320) and transcription factor EB (TFEB) (321), a master regulator of autophagy gene expression. Cardiac TFEB is suppressed in both T1DM and T2DM (322). AKT, mitogen-activated protein kinases 1 and 3 (ERK1/2), and the SIRT family are additional nodal points for autophagy regulation in DCM. AKT (57) and ERK1/2 (323) inhibited autophagic flux in the hearts of obese mice through MTORC1 activation. Strikingly, cardiac *Akt2* knockout preserved cardiac function in high-fat diet-induced obesity by rescuing cardiac autophagosome maturation (301). In contrast, SIRT3 and SIRT1 were downregulated in both STZ-induced (324) and HFD-induced diabetic hearts (325). Consistently, SIRT3 (326) and SIRT1 (327) mediated the cardioprotective effects of resveratrol observed in T1DM by enhancing autophagic flux via activation of FOXO3A (324) and FOXO1 (327). As such, MTORC1, AKT, and ERK1/2 act as negative regulators of autophagy during the development of DCM, while AMPK and the SIRT family are considered as the enhancers.

More recently, non-coding RNAs have been found to be involved in numerous mechanisms underlying the development of DCM, with some of them regulating autophagy. Dysregulation of miRNAs was analyzed in the left ventricle of diabetic mice after STZ-injection, revealing that *mir30a, mir133a, mir212*, and *mir221* are particularly associated with autophagy regulation. Among them, *mir212* and *mir221* were significantly upregulated in diabetic ventricles and even remained increased after insulin treatment (328), suggesting that they are likely involved in cardiac deterioration even with proper glycemic control. *Mir212* targets *Foxo3a* (329), while *mir221* targets *p27* mRNA, modulating MTORC1 activity (330), both of which inhibit autophagy. Additionally, m*ir30d* also targets *Foxo3a* to suppress autophagy in DCM (331). On the other hand, *mir30c* targets *Becn1*, and cardiac overexpression of this miRNA improved cardiac function in genetically obese mice (332), possibly by decreasing BECN1-Rubicon association (292) and improving autophagosome clearance. Lastly, the long non-coding RNA *H19* is downregulated in the heart of STZ-induced diabetic rats, allowing for increased MTORC1 signaling and suppressed autophagy (333). Therefore, emerging evidence demonstrates that non-coding RNA regulation of autophagy also acts as potential therapeutic targets for treating DCM.

### Mitophagy in DCM

Even though mitochondria possess their own UPR, proteotoxic stress or damage can surpass their capacity, requiring a more robust response. Mitochondria can be selectively targeted for autophagy. Without adequate mitochondrial clearance, remaining damaged mitochondria are unable to meet ATP demand, produce excessive ROS, and promote cell death and inflammation (195). Mitochondrial autophagy, termed mitophagy, is directed by the serine/threonine-protein kinase PINK1 (PINK1) phosphorylating the E3 ubiquitin-protein ligase PRKN (PRKN) and fostering mitochondrial protein ubiquitination. The adaptor proteins SQSTM1, neighbor of BRCA1 (NBR1), nuclear dot protein 52 (NDP52), optineurin (OPTN), TAX1BP1 (TBK1), BNIP3, and NIX link damaged mitochondria to autophagosomes for their degradation (334) and can sometimes induce PINK1-independent mitophagy (Figure 6C). PINK1 deletion results in mitochondrial dysfunction, oxidative stress, and cardiomyopathy (335). Similarly, knocking out cardiac PRKN in mice accelerated the development of systolic dysfunction after HFD-feeding, accompanied by the accumulation of dysfunctional mitochondria and lipids (296). ULK1 (336) and RAB9-dependent (312) mitophagy has also been observed in the hearts of diabetic mice, and its impairment aggravated cardiac function. However, the levels of these proteins continued to increase when diastolic (336) and systolic dysfunction (312) were detected; therefore, there is doubt on whether alternative mitophagy could be sufficient to sustain cardiac function during metabolic stress.

Impaired mitophagy is a major contributor to the pathogenesis of DCM. In a compensated stage of HDF feeding, mitophagy is upregulated by the general autophagy ATG7-dependent mechanism, and disrupting mitophagy by deleting cardiac PRKN expression accelerates the appearance of cardiac dysfunction (296). Consistently, PINK1 and PRKN levels were found to be downregulated in the myocardium of STZ-induced and OVE26 diabetic mice with systolic dysfunction (312). Different mechanisms have been proposed to contribute to the loss of mitophagy after metabolic stress. MST1 was found to inhibit cardiac mitophagy in the hearts of diabetic mice via SIRT3- mediated PRKN suppression (196). SIRT3 and PRKN activities were ameliorated by melatonin (337) and icariin administration (338), resulting in improved mitochondrial function. In pancreatic islets and hepatocytes from obese mice, an increase in P53 protein suppressed mitophagy by direct interaction of P53 and PRKN, blunting mitochondrial uptake by autophagosomes (339, 340). Lipid metabolism was associated to HFD-induced PRKN reduction, given that stimulation of lipid catabolism by overexpressing acetyl-coA 2 (ACC2) restored mitophagy and cardiac function in mice (341). Adequate mitochondrial morphology and dynamics are also vital to facilitate mitophagy during DCM. HFD feeding induced dynamin-related protein 1 (DRP1) activity in the hearts of mice and monkeys. DRP1 is the primary regulator of mitochondrial fission, and its increased activity suppressed mitophagy and resulted in cardiac inflammation and heart failure (342, 343). On the contrary, myocardial samples of *ob/ob* mice showed reduced levels of mitofusin 2 (MFN2), the lead regulator of mitochondrial fusion. Restoration of MFN2 expression in cardiomyocytes exposed to high-glucose and high-fat treatment recovered mitochondrial membrane potential and function (344). MFN2 also promotes PRKN translocation and mitophagy in cardiomyocytes (345). Albeit the need to continue exploring the mechanisms governing mitophagy during DCM, its role in maintaining mitochondrial quality has been acknowledged.

## Crosstalk Between PQC Systems

The different PQC systems act as an integrated stress response. They are interconnected and regulate each other at the transcriptional and protein level, and this interdependence is relevant for health and disease. For instance, the UPS downregulates autophagy by processing transcription factors, such as P53, NFkB, HIF, and FOXO (346), and autophagy components, such as BECN1 (347, 348), LC3, p62, and ULK1(346). In cancer cells, chemotherapy resistance to bortezomib, a proteasomal inhibitor, arises from the induction of autophagy as a compensatory mechanism (349). The combination of bortezomib with hydroxychloroquine improved the treatment outcome (350). In turn, proteasomes can be degraded by autophagy. Amino acid starvation-induced autophagic activity also enhances polyubiquitination of 19S regulatory particle, targeting it for autophagic uptake and decreasing proteasomal activity level (351, 352).

Similarly, the UPR^ER^ components, ATF6, CHOP and IRE1 are degraded by the UPS, while two UPR^ER^ branches, PERK-ATF4 and IRE1-XBP1 regulate the expression of UPS components (346). IRE1 is handled through direct interaction with the ERAD complex SEL1-HRD1 and during ER stress, this interaction is broken for UPR^ER^ initiation (353). At the same time, IRE1-XBP1 pathways stimulate the transcription of SEL1L and HRD1 for further UPS function of misfolded proteins (354). This self-modulating feedback loop prevents overactivation and ER-mediated cell death. In *db/db* mice, cardiac expression of HRD1 is reduced, suggesting blunted ERAD activity contributes to prolonged ERSR (355). On the contrary, in doxorubicin-induced cardiomyopathy, UPS activity was observed to be increased (168, 169), perhaps furthering the impairment of UPR^ER^ function (356). Even though the goal of all PQC system is the restoration of protein homeostasis, the impact of each one in cellular function under stress conditions differs.

Clear links between the UPR^ER^, UPS, and autophagy have been acknowledged; however, few of them have been explored in the diabetic heart. Both IRE1 and PERK branches of the UPR^ER^ induce autophagy by promoting BECN1/BCL2 dissociation and upregulating autophagy genes, such as ATG12, BECN1, and LC3. In addition, ER calcium release can stimulate the Ca^2+^/calmodulin-dependent kinase kinase β (CaCMKKβ) that phosphorylates and activates AMPK, resulting in autophagy stimulation (357). UPS malfunction has also been found to provoke autophagy through NRF2-mediated SQSTM1 upregulation (358) and calcineurin-TFEB activation (165), suggesting that autophagy acts as a compensatory mechanism upon proteasomal insufficiency. Proteasomal insufficiency was detected in diabetic hearts previous to cardiac dysfunction (UPS section); therefore, it is possible that these mechanisms contribute to autophagy induction in early stage of DCM. On the other hand, autophagy suppression inhibits UPS function due to the accumulation of SQSTM1 that drives the excessive sequestration of ubiquitinated proteins in protein aggregates, preventing proteasomal degradation (359). Autophagy inhibition in late DCM could be aggravating UPS malfunction and further damaging cardiac function. The exploration of these crosstalks in the heart of diabetes patients and animal models could clarify the status of PQC components and regulatory mechanisms.

## Therapeutics

Proper regulation of the PQC system in the myocardium is vital to maintain cardiac physiology and preserve heart performance in response to pathological stresses. Studies on the alterations and regulation of PQC in the heart in the fact of diabetes mellitus have provided new insights into the molecular pathogenesis of DCM, as well as delivered proof-of-concept evidence that the fine-tuned modulation of the UPR^ER^, UPS, and autophagic event is a potential therapeutic strategy to treat DCM and prevent heart failure in the diabetic populations.

### Targeting the UPR^ER^

As a metabolic disorder, DCM embodies subsequent UPR^ER^ events, where protective ER response is dominant in early stages, followed by decreased UPR^ER^ signaling, and ultimately, irreversible ERSR associated with structural abnormalities in the myocardium (360). Therefore, potentiating initial UPR^ER^ activation to restore protein homeostasis and impeding ER apoptotic response to prevent cell death qualifies as a therapeutic strategy for managing ER stress in DCM (17, 51). Interestingly, exercise, a known physiological UPR^ER^ inducer, mitigated apoptotic ERSR by reducing CHOP and caspase 12 expression, and augmented cardiac function in STZ induced type 1 diabetes (361). This suggests that exercise is beneficial by restoring protein homeostasis.

Modulation of the UPR^ER^ sensors and GRP78 restores cellular homeostasis and improve heart function in multiple cardiovascular disorders. Although GRP78 overexpression is cardioprotective in hypoxia-induced injury (362), normalizing GRP78 is shown to be beneficial for disorders with overactivated UPR^ER^ such as DCM. Moreover, chemically enhancing IRE1-XBP1 (363) and ATF6 activity (364) reduced ER-associated apoptosis following myocardial infarction. Contrastingly, IRE1 and PERK inhibition alleviated atherosclerosis development and decreased cell death in cardiac arrhythmias, respectively. As stated above, PERK and ATF6 hyperactivation are deleterious in the diabetic heart, indicating that our UPR^ER^ knowledge is incomplete in the context of different cardiac etiologies. Nonetheless, the pharmacological modulation of UPR^ER^ signaling following numerous diabetic stresses has dramatically increased in the past few years (Table 2).

**Table 2:** UPR-targeting drugs.

	**Therapeutic application**	**Mechanism/Target**	**Effect on cardiac ER and UPR**	**Type of DM**	**References**
Metformin	Antihyperglycemic	AMPK activation	↑GRP78 ↑PERK/eIF2α ↓ CHOP	T2DM	(365, 366)
Thiazolidinediones	Antihyperglycemic	PPAR activation	↑IRE/XBP1 ↓ IRE/JNK ↓ CHOP	T2DM	(69, 366, 367)
DPP4 inhibitors	Antihyperglycemic	DPP4 inhibition	↓ IRE/JNK ↓ CHOP	T2DM	(366)
GLP1-agonists	Antihyperglycemic	GLP1 receptor activation	↓ CHOP ↓ JNK	T2DMHigh glucose cardiomyocytes	(368–370)
SGLT2 inhibitors	Antihyperglycemic	SGLT2 inhibition	↓ CHOP ↓ Caspase 12	T2DM	(371, 372)
Adiponectin	Appetite and metabolic regulators	Adiponectin receptor activation	↓ IRE/JNK	T2DM	(58)
Angiotensin-II receptor type 1 blockers	Antihypertensive	Angiotensin receptor inhibition	↓ CHOP ↓ GRP78	T1DMT2DM	(373, 374)
Resvertrol	Antioxidant	SIRT activation	↓ PERK/CHOP ↓ ATF6/CHOP ↓ IRE1/JNK	T2DM	(76, 375)
Rapamycin	Antihyperglycemic	UPR inhibition	↓ IRE1/JNK	T2DM	(366)
Tanshinone II	Antioxidant	Superoxide mutase activation	↓ CHOP ↓ GRP78	T1DM	(376)
Matrine	Antioxidant Antiinflammatory	N/A	↓ ATF6 ↓ calreticulin ↓ PERK ↓ GRP78	T1DMT1DM	(61, 377)
Anthocyanins	Antioxidant	Inhibition of oxidation promoting enzymes and ROS scavenging	↓ CHOP ↓ GRP78 ↓ XBP1	T2DM	(378)
Apocyanin	Antioxidant	NAPDH oxidase inhibitor	↓ PERK ↓ GRP78 ↓ ATF6	T1DM	(66)
Melatonin	Antioxidant	Melatonin receptor activator	↓ PERK/CHOP	T2DM	(379)
IL-1 receptor anagonist	Antiinflammatory	IL-1	↓ CHOP	T1DM	(73)
Vanadium deravative	Antihyperglycemic	PPARγ activation	↓ CHOP ↓ GRP78 ↓ XBP1	T2DM	(55)
EGFR inhibitor	Antioxidant	Tyrosine kinase receptor inhibition	↓ CHOP	T1DM/T2DM	(78, 79)
TUDCA	Chemical ER chaperone	GRP78	Binds to misfolded proteins	T2DM/T1DM	(41, 64)
4-PBA	Chemical ER chaperone	N/A	Binds to misfolded proteins	T2DM	(356, 380)

*List of drugs proven to alter cardiac UPR during DCM treatment*.

Poor glycemic control is associated with increased ER stress and decreased function in the diabetic heart. Sodium-glucose cotransporter 2 (SGLT2) inhibitors as an effective-glucose lowering therapy showed robust cardioprotective outcomes in clinical trials (371) and pre-clinical studies (372) by reduced ER-mediated apoptosis following oxidative stress. However, glucagon-like peptide 1 (GLP-1) agonists are shown to exacerbate heart failure or have no significant cardiovascular outcome in T2DM patients (371). GLP-1 agonists are cardioprotective in DCM rodent models by inhibiting UPR^ER^ signaling and ER-mediated apoptosis (368–370, 381). Therefore, these drugs fall short of mitigating heart failure in diabetes patients, possibly due to inhibition of adaptive ERSR. Also, metformin (382) and thiazolidinediones (TZD) (367) lower cardiovascular events in T2DM patients by its antihyperglycemic effects. Meanwhile, in pre-clinical studies, metformin also induced the protective UPR^ER^ function (365, 366), and TZDs improved insulin sensitivity by upstream mediated attenuation of inflammation and ER-associated apoptosis (69), thereby ameliorating cardiac function in diabetes. Taken together, clinically antihyperglycemic drugs fulfil cardioprotective role in DCM, although their function on UPR^ER^ needs to be further confirmed.

Multiple approaches are being employed to improve cardiac function by the administration of anti-ER stress chemicals, which may facilitate UPR^ER^ action (60). Chemical chaperones restored the UPR^ER^, which attenuates maladaptive ERSR under pathological stresses (383), including diabetes (71, 384). 4-phenyl butyric acid (4-PBA) and tauroursdoeoxycholic (TUDCA) improved heart function in doxorubicin-induced cardiomyopathy (356), emphasizing their potential as cardioprotective drugs. Moreover, these chaperones can reduce ER protein load in cardiomyocytes by reducing fatty acid uptake (380) and normalizing GRP78 expression (64) in T2DM rat models. Additionally, TUDCA is currently employed in three clinical trials in amyloid cardiomyopathy associated with the onset of type 1 diabetes (385). Of note, given the ubiquitous nature of UPR^ER^ signaling, these multi-organ targeting drugs may have off-target effects. Therefore, further clarification of specific drug targets is of considerable significance to improve the efficacy of these drugs as DCM therapy.

Other strategies to target ER stress as DCM therapy include antihypertensives, antioxidants, and antiinflammatory compounds. Besides the metabolic alterations, increased angiotensin II signaling in diabetes also induces ER stress in the heart (60). The antihypertensive drugs, such as valsartan, are shown to downregulate CHOP expression and reduce cardiac remodeling in DCM (373, 374). On the other hand, phytochemicals, such as matrine, have attracted attention in attenuating maladaptive ERSR (376) and preserving UPR^ER^ signaling (61, 377), subsequently improving cardiac function in STZ-induced DCM. Moreover, vanadium derivative (55) and endogenous hormones, such as melatonin (379) and FGF21 (386, 387), have been investigated for their cardioprotective role by suppressing oxidative stress-mediated ERSR and cell death in T2DM. Furthermore, targeting upstream regulators of UPR^ER^ signaling, such as SIRT1 (76, 375), might be beneficial as DCM therapy. Therefore, these molecules may be further developed as novel therapeutic agents with clinical efficacy to target UPR^ER^ signaling in DCM. In conclusion, since targeting UPR^ER^ signaling is a two-edged sword, proper UPR^ER^ regulation is essential to restore protein homeostasis in the cardiomyocytes, while inappropriate suppression of ERSR may have unpredictable effects on cardiac function in DM populations.

### Targeting the UPS

#### Targeting the Proteasome

Given its indispensable role in maintaining cellular proteostasis, the proteasome is a potent therapeutic target to treat proteotoxic stress in the heart. Benign enhancement of proteasomal function by overexpression of the 11S proteasomal subunit PA28α markedly reduced aberrant protein aggregation and cardiac hypertrophy in a mouse model of *CryAB*^*R*120*G*^ proteinopathy (388). Likewise, cardiac-specific proteasome enhancement partially improved right ventricular dysfunction and survival in mice subjected to pressure overload (389). More recently, Li et al. reported that restoration of proteasome function facilitated by PA28α overexpression preserves cardiac hemodynamics and ameliorates diabetes-induced pathological cardiac remodeling in STZ-induced diabetic mice (222). These salient findings suggest that genetic proteasome enhancement restores PQC and improves cardiac function in response to various pathological conditions, including metabolic stress.

Pharmacological stimulation of cAMP-PKA and cGMP-PKG signaling by phosphodiesterase (PDE) inhibitors can also activate cardiac proteasome activity. The synthesis of cAMP and cGMP is mediated by adenylyl cyclases or guanylyl cyclases, respectively, whereas their degradation is mediated by PDEs (149). Thus, inhibiting PDEs increases cellular levels of cAMP and cGMP. Eleven PDE families have been identified; among them, PDE1, PDE2, PDE3, PDE4, PDE5, and PDE8 are expressed in the heart (390). Ranek et al. (391) demonstrated that sildenafil, an FDA approved PDE5 inhibitor functioning on activation of PKG, stimulates proteasome peptidase activity, enhances the clearance of misfolded proteins, and decreases aberrant protein aggregation, thereby improving cardiac proteostasis. More recently, it has been reported that pharmacological inhibition of PDE1 (IC86430) increases cardiac proteasome function and accelerates proteasomal degradation of aberrant myocardial proteins in a PKA- and PKG-mediated manner (392). Strikingly, the administration of IC86430 at an overt disease stage markedly improved diastolic function and delayed premature death in *CryAB*^*R*120*G*^ mice (392). Conclusively, pharmacological enhancement of proteasome activity by stimulating PKA or PKG is likely a novel strategy to treat DCM by eliminating aggregation of damaged proteins and alleviating cellular proteotoxicity.

#### Targeting the E3 Ubiquitin Ligases

Due to their ability to regulate UPS, the E3 ubiquitin ligases represent promising drug targets for patients with diabetic heart disease. Hydrogen sulfide (H_2_S) is a gastotransmitter to maintain cardiovascular homeostasis, which is blunted in various cardiovascular diseases, including DCM (355). H_2_S primarily signals through a specific protein modification termed sulfhydration, whereby the thiol group of a reactive cysteine is converted to an hydropersulfide (–SSH) group (393, 394). Recent work by Yu et al. (355) has shown that exogenous H_2_S reduces translocation of the free fatty acid (FFA) transporter CD36 from intracellular stores to the plasma membrane by promoting sulfhydration of the ER-resident ubiquitin ligase, HRD1; thereby attenuating myocardial fatty acid uptake and lipotoxicity in *db/db* mice. More specifically, HRD1 S-sulfhydration regulates the ubiquitylation of VAMP3 (involved in CD36 trafficking) and promotes its degradation. Interestingly, H_2_S-generating compounds have been tested in various preclinical models of heart disease (395). For instance, SG-1002, an orally active H_2_S prodrug, attenuates cardiac dysfunction in HFD-induced type II diabetic mice (394). Of note, SG-1002 has been clinically investigated in patients with cardiovascular disease (396). Thus, H_2_S may hold therapeutic potential for the treatment of DCM.

Additionally, AMPK has been shown to regulate the transcription of two ubiquitin ligases in the heart; Atrogin-1 and MURF1 (397). AMPK activation leads to increased rates of UPS-mediated protein degradation, thereafter increasing amino acid availability for protein synthesis or ATP production as the heart adapts to a deteriorating metabolic milieu (397). In the diabetic heart, AMPK-mediated protein lysis is cardioprotective due to preserved PQC (22, 397). Numerous studies have reported that metformin improves clinical outcomes in patients with diabetic heart failure by activating AMPK (398–400); however, whether and how metformin regulates cardiac UPS in the progression of DCM requires further investigation.

### Targeting Autophagy

Autophagy has been targeted for the treatment of cardiovascular disease; however, in DCM, it is not yet determined whether the induction or inhibition of autophagy has potential treatment effects. Preventive and therapeutic advice in diabetes includes lifestyle modifications, such as exercise and caloric restriction, which have been shown to induce cardiac autophagy and diminish the risk of cardiac events (401). Caloric restriction is sometimes supported by the prescription of appetite suppressors, among which adiponectin (302, 402), leptin (403), and GLP1 receptor agonists (404, 405) have also been proved to induce cardiac autophagy in diabetic models.

Pharmacological treatment for DCM relies mostly on the attenuation of the systemic derangements that lead to cardiac stress; nevertheless, several of these treatments are also able to activate autophagy in cardiac cells (Table 3). As mentioned above, one of the most widely prescribed insulin sensitizers is metformin. This AMPK activator improved cardiac function in animal DCM (315) and human heart failure (400). Similarly, improving glycemic control by other insulin sensitizers (302, 408–412), stimulating insulin secretion (413, 414, 416–418), or reducing glucose uptake in the renal tube (419–421) has cardioprotective effects, also restoring myocardial autophagy in DCM models. Lipid-lowering treatments (422, 423) and antihypertensives (424, 428, 452) enhanced myocardial autophagy in preclinical models of T1DM and T2DM through different molecular mechanims, such as calcium-mediated autophagosome-lysosome fusion and receptors modulation. Despite some conflicting results (453), the antioxidant supplements, resveratrol (327, 432, 433, 454, 455), spermidine (437–440), and epigallocatechin gallate (EGCG) (441–444) protected the heart in clinical and preclinical studies in both types of diabetes, also by regulating myocardial autophagy. While the systemic effects of these treatments are essential, the cardiac-specific regulation of autophagy is necessary for the maintenance of cardiac function in diabetes. Further research is required to delineate which drugs in each class are the most beneficial.

**Table 3:** Autophagy-targeting drugs.

**Drug**	**Therapeutic application**	**Mechanism/Target**	**Effect on cardiac autophagy**	**Type of DM**	**References**
Adiponectin	Appetite and metabolic regulators	Adiponectin receptor activation	Induction	T2DM	(302, 402)
Leptin	Appetite and metabolic regulators	Leptin receptor activation	Induction	T2DM	(403, 406)
Metformin	Antihyperglycemic	AMPK activation	Induction	T1DM/T2DM	(315, 316, 400, 407)
Rapamycin and analogs	Antihyperglycemic	MTORC1 inhibition	Induction	T2DM	(302, 408, 409)
Thiazolidinediones	Antihyperglycemic	PPAR activation	Induction	T2DM/T1DM	(410–412)
DPP4 inhibitors	Antihyperglycemic	DPP4 inhibition	Induction	T2DM	(413–415)
GLP1-agonists	Antihyperglycemic	GLP1 receptor activation	Induction	T2DM	(404, 416–418)
SGLT2 inhibitors	Antihyperglycemic	SGLT2 inhibition	Induction	T2DM	(419–421)
Fenofibrate	Lipid-lowering	PPARα activation	Induction	T1DM	(422)
Statins	Lipid-lowering	HMG-CoA reductase inhibition	Induction	T2DM	(423)
Verapamil	Antihypertensive	Calcium channel inhibition	Induction	T1DM/T2DM	(424, 425)
Nifedipine	Antihypertensive	Calcium channel inhibition	Induction	T1DM	(426, 427)
Valsartan	Antihypertensive	Angiotensin receptor inhibition	Induction	T1DM/T2DM	(428–431)
Resveratrol	Antioxidants	SIRT activation	Induction	T1DM/T2DM	(327, 432–436)
Spermidine	Dietary supplement	Acetylase inhibitor	Induction	T1DM	(437–440)
EGCG	Antioxidant and antiinflammatory	SIRT1 activation	Inhibition	T1DM/T2DM	(441–444)
Suberoylanilide hydroxamic acid (SAHA)	Antineoplastic	HDAC inhibition	Induction	T1DM	(445)
Granulocyte-colony stimulating factor (GCSF)	Hematopoietic cytokine	GCSF receptor activation	Inhibition	T2DM	(446, 447)
Hydroxychloroquine	Antimalarial and immunosuppressive	Lysosomal inhibition	Inhibition	T2DM	(448–451)

*List of drugs proven to affect cardiac autophagy during DCM treatment*.

Some nondiabetic treatments also display benefits in cardiovascular health in diabetes through autophagy regulation. Suberoylanilide hydroxamic acid (SAHA), a histone deacetylase inhibitor, restored cardiomyocyte contractility in STZ-injected rats (445) and can promote cardiac autophagy (446). On the other hand, the granulocyte-colony stimulating factor (GCSF) stimulates bone marrow function and ameliorated diastolic dysfunction in rodent T2DM models (447, 456, 457) by downregulating autophagy (448). The antimalarial drug and lupus treatment hydroxychloroquine is mostly associated with high risks of cardiotoxicity and heart failure (458); nevertheless, it improved β-cell function in obese nondiabetic patients (449) and reduced glycemic levels in T2DM patients (450). Hydroxychloroquine represses autophagy by accumulating in lysosomes and inhibiting autophagosome fusion (459). Considering this, it remains to be explored whether reduced autophagy might be a therapeutic strategy in early stages of disease and whether targeting cardiac autophagy is an adjuvant strategy for the current metabolic treatments to prevent DCM and heart failure in the diabetic populations.

## Conclusions

**Figure 8:**
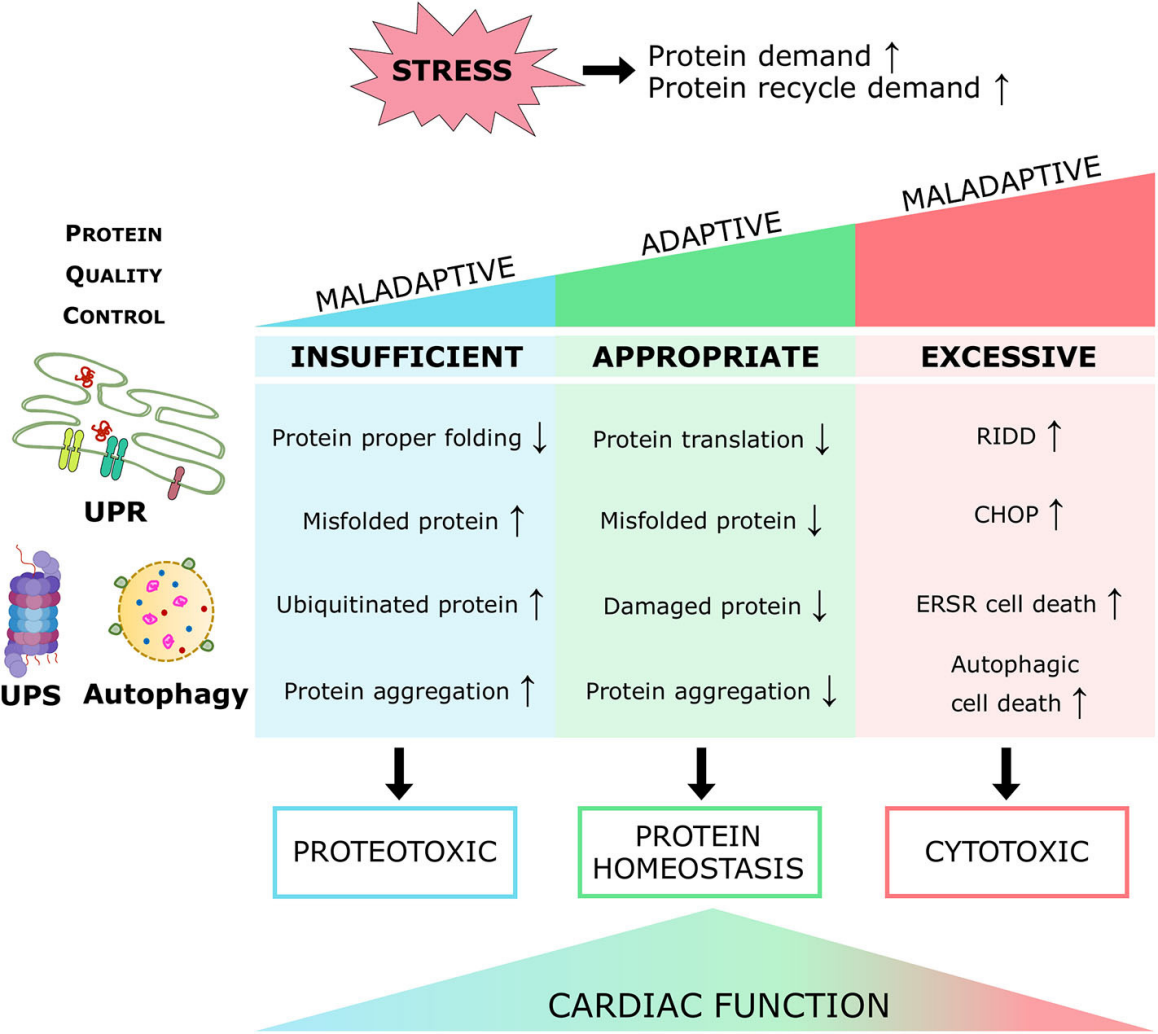
Role of PQC systems in cardiac function under pathological stress. Either insufficient or excessive activity of the main PQC systems is maladaptive. By inducing protein or cellular toxicity, they contribute to cardiac dysfunction. Appropriate level of PQC maintains cardiac protein homeostasis and sustains cardiac function.

Accumulating evidence on the molecular pathogenesis of DCM has revealed the essential roles of proper cellular protein quality control in diabetes-associated heart disease. Upon diabetic stress, the PQC machinery senses, detects, and disposes of the damaged proteins by multiple processes, including the UPR, the UPS, and autophagy. Concerted action of the three cellular systems can tackle proteotoxicity, subsequently improving the cardiac outcome in diabetes; accordingly, compromised PQC mechanisms appear to contribute to heart disease as a result of impaired cellular homeostasis. Dysregulation of the UPR results in the accumulation of misfolded proteins, while malfunction of the UPS and autophagy lead to aggregation of toxic proteins in the cytosol, all of which triggers cell death and provokes the onset and development of heart failure in diabetes. Therefore, it is suggested that the maintenance of protein homeostasis may be a valuable and promising therapeutic strategy to treat cardiac complications in diabetes patients. Of note, either insufficient or exaggerated action of the principal mechanisms exacerbates cytotoxicity in the face of pathological stresses, including diabetic stress (Figure 8). Given this dual role in the heart, finely tuned manipulation of the UPR, the UPS, and autophagy in the myocardium is mandatory to maintain cellular equilibrium in response to the diabetic condition with increased demand of protein turnover. As such, despite the growing knowledge of the mechanisms underlying cardiac proteostasis networks, further research is indispensable to investigate the therapeutic potential for heart disease by targeting PQC molecules in diabetes mellitus. As the understanding of molecular regulation of PQC function develops, so will our capability to exploit pharmacological interventions to prevent proteotoxicity and cardiac dysfunction in diabetic populations.

## Author Contributions

NK, RR, and AR-V collected references, generated tables and figures, and drafted the manuscript. AR-V and WL designed the work, wrote, and proofread the manuscript. All authors contributed to the article and approved the submitted version.

## Conflict of Interest

The authors declare that the research was conducted in the absence of any commercial or financial relationships that could be construed as a potential conflict of interest.
